# Proteomic and Transcriptomic Analyses Provide Novel Insights into the Crucial Roles of Host-Induced Carbohydrate Metabolism Enzymes in *Xanthomonas oryzae* pv. *oryzae* Virulence and Rice-*Xoo* Interaction

**DOI:** 10.1186/s12284-021-00503-x

**Published:** 2021-06-26

**Authors:** Guichun Wu, Yuqiang Zhang, Bo Wang, Kaihuai Li, Yuanlai Lou, Yancun Zhao, Fengquan Liu

**Affiliations:** 1 Jiangsu Key Laboratory for Food Quality and Safety-State Key Laboratory Cultivation Base of Ministry of Science and Technology, Institute of Plant Protection, Jiangsu Academy of Agricultural Sciences, No. 50 Zhongling Street, Nanjing, Jiangsu 210014 P. R. China; 2 State Key Laboratory of Microbial Technology, Marine Biotechnology Research Center, Shandong University, Qingdao, 266237 P. R. China; 3 Key Laboratory of Integrated Management of Crop Diseases and Pests, Ministry of Education, College of Plant Protection, Nanjing Agricultural University, Nanjing, 210095 P. R. China

**Keywords:** *Xoo*-rice interaction, Host-induced proteins, Carbohydrate metabolism enzyme, Pathogenicity, Differentially expressed genes

## Abstract

**Background:**

*Xanthomonas oryzae* pv. *oryzae* (*Xoo*) causes bacterial leaf blight, a devastating rice disease. The *Xoo*-rice interaction, wherein wide ranging host- and pathogen-derived proteins and genes wage molecular arms race, is a research hotspot. Hence, the identification of novel rice-induced *Xoo* virulence factors and characterization of their roles affecting rice global gene expression profiles will provide an integrated and better understanding of *Xoo*-rice interactions from the molecular perspective.

**Results:**

Using comparative proteomics and an in vitro interaction system, we revealed that 5 protein spots from *Xoo* exhibited significantly different expression patterns (|fold change| > 1.5) at 3, 6, 12 h after susceptible rice leaf extract (RLX) treatment. MALDI-TOF MS analysis and pathogenicity tests showed that 4 host-induced proteins, including phosphohexose mutase, inositol monophosphatase, arginase and septum site-determining protein, affected *Xoo* virulence. Among them, mutants of two host-induced carbohydrate metabolism enzyme-encoding genes, Δ*xanA* and Δ*imp*, elicited enhanced defense responses and nearly abolished *Xoo* virulence in rice. To decipher rice differentially expressed genes (DEGs) associated with *xanA* and *imp*, transcriptomic responses of Δ*xanA*-treated and Δ*imp*-treated susceptible rice were compared to those in rice treated with PXO99^A^ at 1 and 3 dpi. A total of 1521 and 227 DEGs were identified for PXO99^A^ vs Δ*imp* at 1 and 3 dpi, while for PXO99^A^ vs Δ*xanA*, there were 131 and 106 DEGs, respectively. GO, KEGG and MapMan analyses revealed that the DEGs for PXO99^A^ vs Δ*imp* were mainly involved in photosynthesis, signal transduction, transcription, oxidation-reduction, hydrogen peroxide catabolism, ion transport, phenylpropanoid biosynthesis and metabolism of carbohydrates, lipids, amino acids, secondary metabolites, hormones, and nucleotides, while the DEGs from PXO99^A^ vs Δ*xanA* were predominantly associated with photosynthesis, signal transduction, oxidation-reduction, phenylpropanoid biosynthesis, cytochrome P450 and metabolism of carbohydrates, lipids, amino acids, secondary metabolites and hormones. Although most pathways were associated with both the Δ*imp* and Δ*xanA* treatments, the underlying genes were not the same.

**Conclusion:**

Our study identified two novel host-induced virulence factors XanA and Imp in *Xoo*, and revealed their roles in global gene expression in susceptible rice. These results provide valuable insights into the molecular mechanisms of pathogen infection strategies and plant immunity.

**Supplementary Information:**

The online version contains supplementary material available at 10.1186/s12284-021-00503-x.

## Background

Plants and pathogens have engaged in arm races for millions of years. As a result of this struggle, complex recognition and defense mechanisms such as pathogen-associated molecular pattern-triggered immunity (PTI), effector-triggered immunity (ETI), accumulation of phytoalexins, reinforcement of plant cell walls, production of reactive oxygen species (ROS) and antimicrobial peptides and synthesis of pathogenesis-related (PR) proteins have evolved in plants to prevent or reduce infection by pathogens (Dodds and Rathjen 2010). Simultaneously, the response of pathogens to plant defenses is also complex and sophisticated, involving a battery of biological and physiological processes, represented by secretion of effectors, activation of virulence factors, modification of host gene expression and evolution of pathogenic strategies to evade host immune attacks (Ryan et al. 2011, Morris et al. 2017). Thus, revealing more molecular signatures of plant-pathogen interactions will enable us to develop effective strategies for the control of plant disease outbreaks with benefits for crop yields and food security.

*Xanthomonas oryzae* pv*. oryzae* (*Xoo*), the causative agent of bacterial blight of rice, is one of the model organisms for studying the molecular mechanisms of plant-bacterium interactions and causes serious reductions in rice yields worldwide. The pathogenicity of *Xoo* and most other *Xanthomonas* pathogens largely depends on the coordinated expression of virulence genes and regulatory systems, including virulence-associated protein secretion systems (type I to type VI) and their substrates, the quorum sensing system (QS), the two-component signal transduction system (TCSs), the cyclic di-GMP signaling pathway, the Csr/Rsm posttranscriptional system and some well-characterized transcriptional factors (Ryan et al. 2011). In addition, several host-induced genes/proteins involved in plant-bacterium interactions have also been revealed by a variety of comparative transcriptomic and proteomic studies in vivo and in vitro. For example, genes related to adhesion, plant cell-wall degradation and insertion sequence (IS) elements of an African *Xoo* strain MAI1 (Soto-Suarez et al. 2010), proteins associated with nutrient uptake, protease/peptidase, and host defense and genes encoding transposases, EF-Tu, the TAL effector and carbohydrate metabolism-related proteins of Asian *Xoo* strain K3 (Wang et al. 2013, Lee et al. 2017) were found to be differentially or specifically expressed during in planta infection (the in vivo host-pathogen interaction). Moreover, during in vitro culture conditions (in an in vitro assay system), host leaf extract (HLX) was successfully used to simulate the interactions between *Xanthomonas* species and their hosts, and a relevant proteomic study showed that *Xap* (*Xanthomonas axonopodis* pv. *passiflorae*) increased the abundance of several crucial proteins (inorganic pyrophosphatase, XadA and YciF) for infection in response to Passiflora leaf extract (PLX) (Tahara et al. 2003). It was also observed in *Xoo* that the expression of genes related to ion transport, chemotaxis and pathogenicity could be induced upon initial interactions with rice leaf extract (RLX) (Kim et al. 2016). However, the functions of host-induced proteins or genes in the *Xoo*-rice interaction have rarely been studied.

As the most widely consumed staple food crop, rice (*Oryza sativa*) is frequently attacked by bacterial, viral or fungal diseases. Among these rice diseases, bacterial blight is one of the major limiting factors of rice productivity, and many studies have provided proteomic and transcriptomic analyses of the rice response to *X*. *oryzae* infection, which may contribute to understanding the molecular mechanism of rice-*X*. *oryzae* interactions. For example, proteomic analysis of rice plasma membrane fractions at 12 and 24 h after *Xoo* inoculation revealed that 11 proteins, including H^+^-ATPase, protein phosphatase, OsHIR1, OsPHB2, zinc finger domain protein, universal stress protein (USP), and heat shock protein, were differentially regulated between the incompatible and compatible interactions mediated by Xa21 (Chen et al. 2007). Furthermore, comparative proteomics revealed that proteins related to photosynthesis, signal transduction and antioxidant defense in somatic hybrid rice (Yu et al. 2008) and biotic and abiotic stress response-associated proteins (such as germin-like proteins and host defense proteins) in resistant rice genotypes were induced during *Xoo* infection (Kumar et al. 2015). Likewise, in the early defense responses of rice after *Xoo* inoculation, genes related to cell signaling, transcription, growth and basal metabolic components were largely found to be differentially expressed in resistant rice compared to susceptible rice (Grewal et al. 2012). In the interacting transcriptomes between rice and *Xoo*, rice genes involved in signal transduction, regulation and resistance were upregulated in the incompatible interaction of rice H471 compared with that of its parents (Zhang et al. 2015). Jha et al. revealed that a number of genes related to defense and stress were upregulated, while those related to metabolism and transport were downregulated following *Xoo* ClsA treatment (Jha et al. 2010). Notably, Lee et al. performed transcriptomic analysis of *Xoo* under different in planta growth conditions and revealed detailed information on differentially regulated genes between susceptible and resistant host-*Xoo* interactions (Lee et al. 2017). In the dual RNA-Seq of *Xanthomonas oryzae* pv. *oryzicola* (*Xoc*) infecting rice, the T3SS defective (T3SD) strain transcriptome in planta was characterized by differential regulation of ATP, protein, polysaccharide synthesis, antioxidation and detoxification related genes, and rice inoculated with T3SD strain resulted in significant expression changes of a series of plant defence related genes (Liao et al. 2019). These previous studies have considerably enhanced our knowledge of the interactions between different rice cultivars and *Xanthomonas oryzae* strains; however, there are still several aspects of rice-*Xoo* interaction such as identification of novel host-induced virulence factors and investigating their roles in rice gene expression profiles yet to be elucidated.

Therefore, in this study, a comparative proteomics approach in accordance with an in vitro interaction system was performed to identify *Xoo* differentially expressed proteins at 3, 6 and 12 h after susceptible rice leaf extract (RLX) treatment, and this led to the identification of 4 host-induced proteins, including phosphohexose mutase (XanA), inositol monophosphatase (Imp), arginase (RocF) and septum site-determining protein (MinD), that were involved in *Xoo* virulence. Then, we further investigated the comparative transcriptomes in susceptible rice IR24 inoculated with wild-type strain PXO99^A^ relative to those of Δ*xanA* and Δ*imp* (2 mutants of host-induced carbohydrate metabolism enzyme-encoding genes) at 1 and 3 days post inoculation (dpi) to understand the genome-wide transcriptional responses of infected rice types that exhibit significantly different disease symptoms. The results obtained in this study reveal the potential functions of host-induced carbohydrate metabolism enzymes in *Xoo*-rice interactions and provide novel insights into the molecular basis of the rice response to *Xoo* infection.

## Results

### Identification of Host-Induced Proteins of *X*. *oryzae* pv. *oryzae* PXO99^A^ in the In Vitro Assay System Using IR24 RLX

**Fig. 1:**
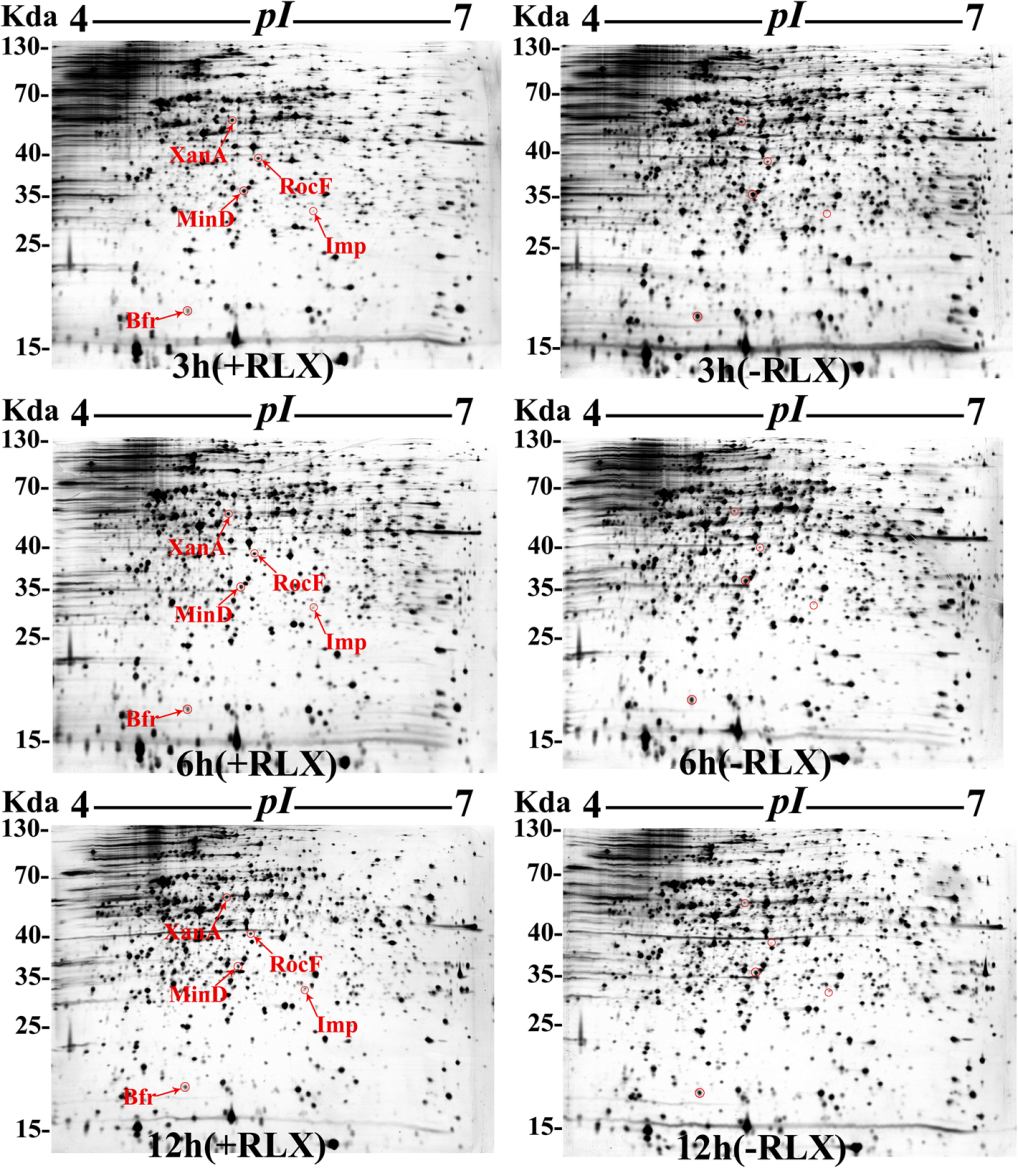
Identification of host-induced proteins of *X*. *oryzae* pv. *oryzae* PXO99^A^ in the in vitro assay system using comparative proteomic analysis. Representative 2-DE profiles of the total proteins of *X*. *oryzae* pv. *oryzae* at 3, 6, and 12 h after treatment with NB plus rice leaf extract (+RLX; left gel) and NB (−RLX; right gel). Protein spots that were significantly altered (|fold change| > 1.5) in +RLX groups compared to -RLX groups at all three time points are indicated by red arrows and circles. These protein spots were excised from silver-stained gels and identified via MALDI-TOF-MS. Detailed information regarding 5 successfully identified proteins was provided in Table 1. The experiments were repeated three times independently, with similar results

To identify proteins of *X*. *oryzae* pv. *oryzae* PXO99^A^ that were up- or downregulated during its interaction with host rice IR24, an in vitro assay system combined with two-dimensional gel analysis was used to compare the total protein expression profiles from RLX-treated and untreated *Xoo* cells. RLX was added at the early exponential phase (OD_600_ ≈ 0.3) of *Xoo* cell culture in NB medium. After 3, 6 and 12 h of treatment, samples from +RLX (NB medium plus rice leaf extract) and -RLX (NB medium) were harvested and distinguished by two-dimensional electrophoresis (Fig. 1). When compared with the control groups (−RLX), 5 protein spots with more than 1.5-fold differential expression were detected at all three time points. Further analysis of these 5 protein spots by MALDI TOF MS and NCBI BLAST led to the identification of 5 unique proteins, including phosphohexose mutase XanA (PXO_03174), arginase RocF (PXO_02850) and inositol-1-monophosphatase Imp (PXO_00388), which were significantly upregulated, and bacterioferritin Bfr (PXO_01151) and septum site-determining protein MinD (PXO_04464), which were significantly downregulated (Table 1). In this study, we refer to these continuously differentially expressed proteins as host-induced proteins.

**Table 1:** Proteins exhibited significantly different expression patterns (|fold change| > 1.5) in *X*. *oryzae* pv. *oryzae* at all three time points after susceptible rice leaf extract (RLX) treatment

Protein name^**a**^	Protein code^**a**^	Accession no.^**a**^	3 h Fold change (+RLX/−RLX)^**b**^	6 h Fold change (+RLX/−RLX)^**b**^	12 h Fold change (+RLX/−RLX)^**b**^	Function/Similarity^**c**^	Functional catalog^**c**^	Predicted cellular localization^**d**^	pI (cal)^**e**^	***M***_**w**_ (cal) kDa^**e**^
XanA	PXO_03174	gi|188,523,095	+ 2.77	+ 1.60	+ 2.76	Phosphohexose mutase	Carbohydrate transport and metabolism	Cytoplasmic; Periplasmic	5.19	49.13
RocF	PXO_02850	gi|188,523,421	+ 3.74	+ 3.22	+ 8.85	Arginase	Amino acid transport and metabolism	Cytoplasmic	5.28	33.36
Imp	PXO_00388	gi|188,520,635	+ 1,000,000	+ 3.37	+ 1.69	Inositol-1-monophosphatase	Carbohydrate transport and metabolism	Cytoplasmic	7.62	30.27
Bfr	PXO_01151	gi|188,577,142	−2.24	−1.63	−1.71	Bacterioferritin	Inorganic ion transport and metabolism	Cytoplasmic	4.93	18.73
MinD	PXO_04464	gi|188,519,996	−1.69	−1.96	−1.88	Septum site-determining protein MinD	Cell cycle control, cell division, chromosome partitioning	Cytoplasmic; innermembrane	5.32	28.93

^a^The protein name, protein code and accession number of identified proteins were according to genomic annotation of *X*. *oryzae* pv. *oryzae* PXO99^A^

^b^The average fold change in NB medium plus rice leaf extract (+RLX) compared to NB medium (−RLX)

^c^The similarity and functional catalog were performed by using protein blast (http://blast.ncbi.nlm.nih.gov/Blast.cgi) and eggNOG 4.5 (http://eggnogdb.embl.de/)

^d^Bacterial protein subcellular localization prediction was performed by PSORTb v.3.0 (http://www.psort.org/psortb/)

^e^Computation of the theoretical pI (isoelectric point) and Mw (molecular weight) for identified proteins was performed by expasy tool (http://web.expasy.org/compute_pi/)

### Evaluation of the Role of Host-Induced Proteins in *X*. *oryzae* pv. *oryzae* PXO99^A^ Virulence in Rice

**Fig. 2:**
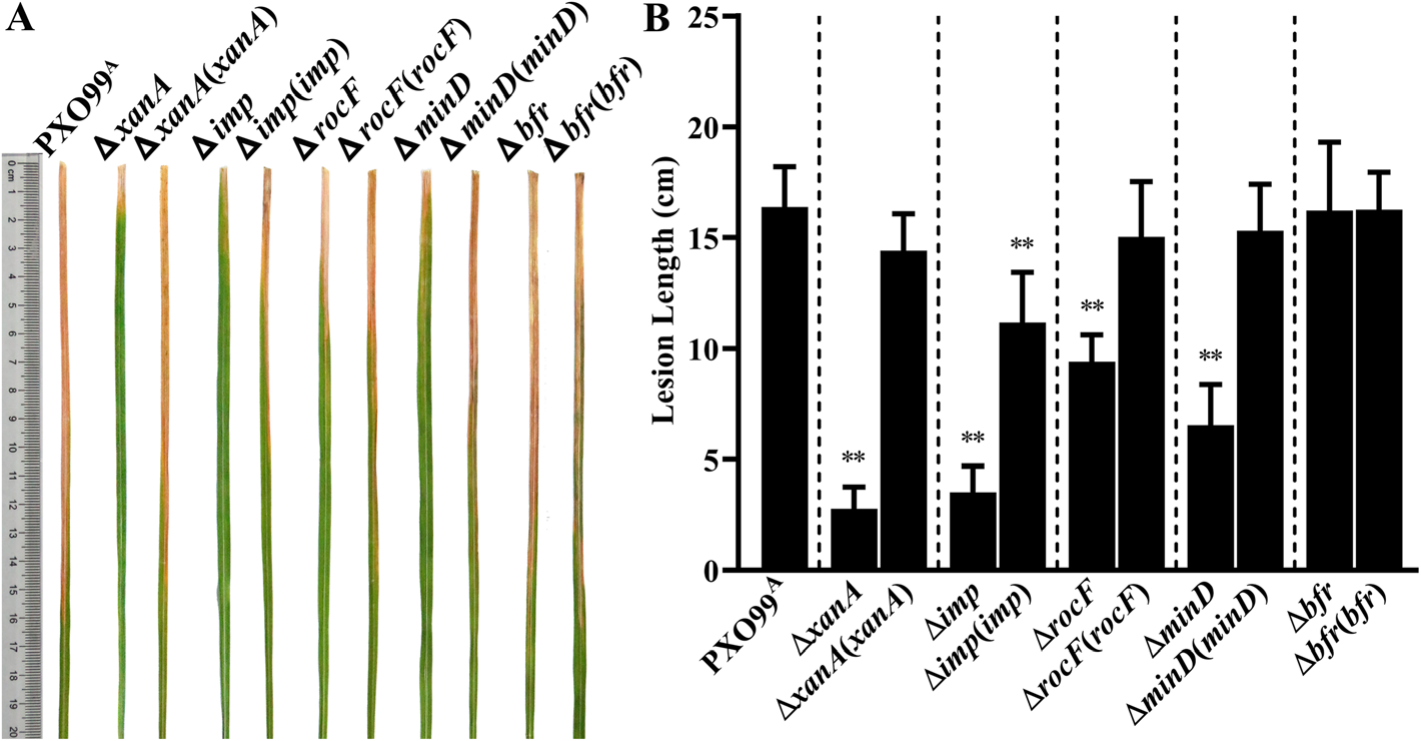
Four of 5 host-induced proteins were involved in *X*. *oryzae* pv. *oryzae* PXO99^A^ virulence on susceptible rice plants. **A** Inoculation of PXO99^A^, Δ*xanA*, Δ*imp*, Δ*rocF*, Δ*minD*, Δ*bfr* and their complemented strains onto the susceptible rice IR24 by the leaf clipping method. Representative disease symptoms were recorded 16 dpi (days post inoculation). **B** Lesion length caused by tested strains on leaves of susceptible rice IR24 at 16 dpi. Values are the means ± standard deviation (SD) from three independent experiments. Asterisks indicate significant differences compared with wild-type (t-test, **P < 0.01). PXO99^A^ is wild-type *X. oryzae* pv. *oryzae*; Δ*xanA*, the *xanA* deletion mutant; Δ*xanA*(*xanA*), the complemented strain of Δ*xanA*; Δ*imp*, the *imp* deletion mutant; Δ*imp*(*imp*), the complemented strain of Δ*imp*; Δ*rocF*, the *rocF* deletion mutant; Δ*rocF*(*rocF*), the complemented strain of Δ*rocF*; Δ*minD*, the *minD* deletion mutant; Δ*minD*(*minD*), the complemented strain of Δ*minD*; Δ*bfr*, the *bfr* deletion mutant; Δ*bfr*(*bfr*), the complemented strain of Δ*bfr*

It was of interest to determine whether host-induced proteins are essential for causing bacterial blight. Thus, we generated in-frame deletion mutants of the genes encoding 5 host-induced proteins in *X*. *oryzae* pv. *oryzae* PXO99^A^ by using the suicide vector pK18mobsacB. The virulence of PXO99^A^, the derived mutants (∆*xanA*, ∆*rocF*, ∆*imp*, ∆*minD* and ∆*bfr*) and their complemented strains were evaluated by performing pathogenicity tests on the susceptible rice IR24 as described in the Materials and Methods. The disease symptoms and lesion lengths were scored 16 dpi. As shown in Fig. 2, when compared with the wild type PXO99^A^, no obviously different disease symptoms or lesion lengths were observed on the leaves inoculated with ∆*bfr*. However, the average lesion lengths after infection with ∆*xanA*, ∆*imp*, ∆*rocF* and ∆*minD* were 2.79 ± 0.98, 3.52 ± 1.18, 9.41 ± 1.22 and 6.54 ± 1.84 cm, respectively, which were significantly shorter than those of the WT (16.38 ± 1.83 cm, *P* < 0.01). Plasmid-based in trans complementation fully or partially restored the deficiency of each mutant in virulence to WT levels. These results demonstrated that 4 of the 5 host-induced proteins were required for the full virulence of PXO99^A^, indicating their important roles in the infection process.

Next, to explore whether the growth ability of ∆*xanA*, ∆*imp*, ∆*rocF* and ∆*minD* contributed to their impaired virulence, growth assays were conducted in NB medium. As shown in Figure S1, all tested mutants displayed a WT growth pattern in NB medium, whereas ∆*imp* and ∆*minD* showed decreased growth ability compared with those of the WT and their respective complemented strains. These results suggested that mutation in genes *imp* and *minD* impaired the fitness of *X*. *oryzae* pv. *oryzae*, thus representing at least one of the mechanisms underlying their involvement in virulence.

### Cellular Defense Responses of Rice Leaves when Infected with PXO99^A^ and Mutants of Host-Induced Carbohydrate Metabolism Enzyme Encoding Genes

**Fig. 3:**
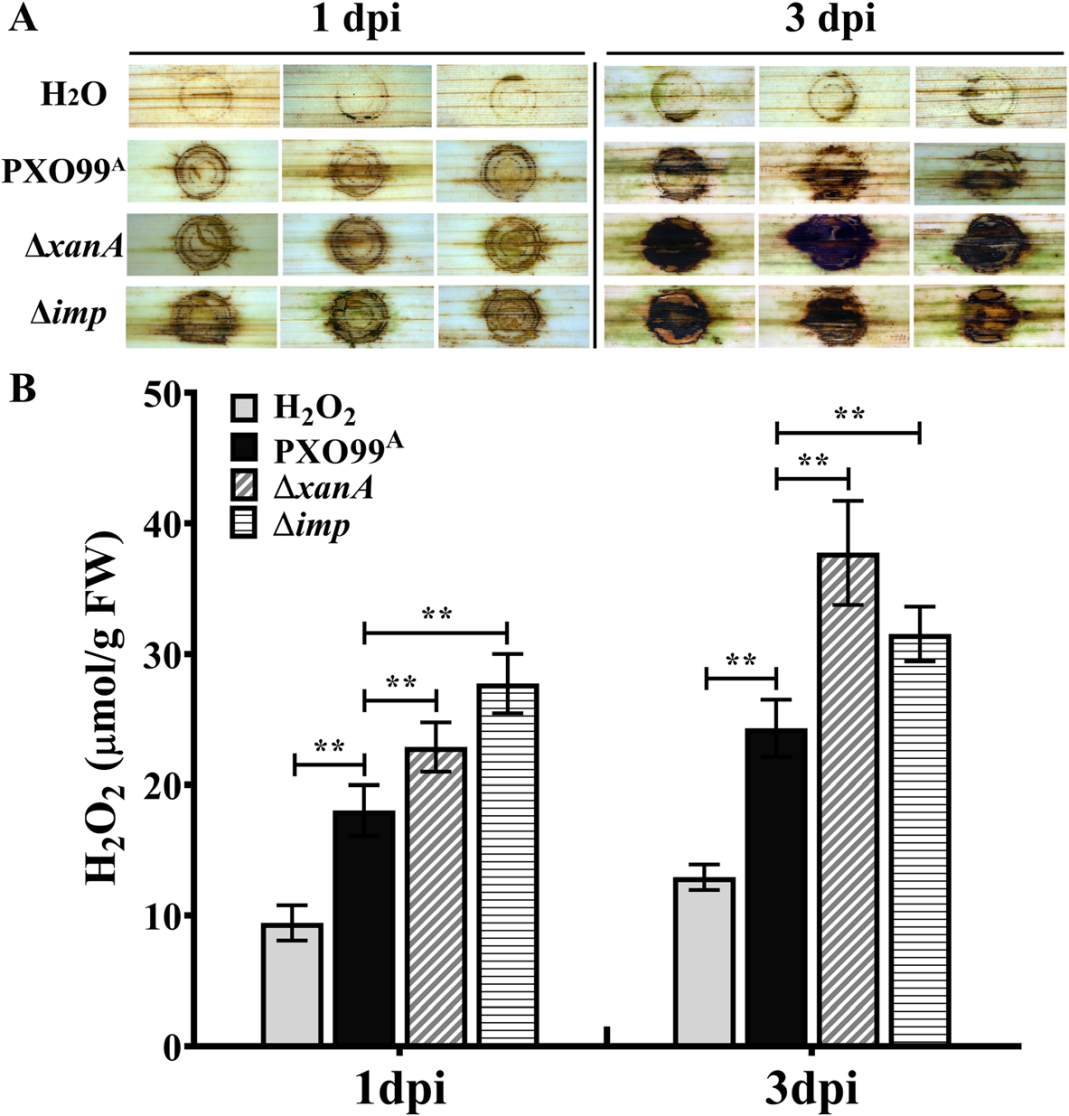
Detection and assessment of cellular defense responses in rice IR24 at 1 and 3 d after infection with PXO99^A^, Δ*xanA* or Δ*imp*. **A** DAB staining and visualization of hydrogen peroxide in IR24 rice. 5-week-old rice leaves were detached and infiltrated with DAB staining solution (1 mg/mL, pH 3.8) used for oxidative burst detection after inoculation with H_2_O, PXO99^A^, Δ*xanA* and Δ*imp* via 1-ml needleless syringes. PXO99^A^-inoculated leaves were used as controls. Orange-brown deposits were visualized after the leaves were cleared in absolute ethanol. Representative photos were taken at 1 and 3 dpi. **B** Quantification of H_2_O_2_ levels in rice leaves after inoculation with H_2_O, PXO99^A^, Δ*xanA* and Δ*imp* via 1-ml needleless syringes. Values are the means and standard deviation of three independent experiments, each of which comprised two replicates. Asterisks indicate significant differences compared with controls (t test, ** indicates *P* < 0.01)

We noticed that *Xoo* almost completely lost virulence when two carbohydrate metabolism enzyme encoding genes *xanA* (phosphohexose mutase) or *imp* (inositol-1-monophosphatase) were knocked out. To determine whether the dramatically reduced virulence of ∆*xanA* and ∆*imp* might be due to greater elicitation of rice defense responses, the oxidative burst, a typical landmark event of cellular defense response, was detected using 3,3-diaminobenzidine (DAB) staining and a Hydrogen Peroxide Assay Kit as described in the Methods section. The orange-brown deposits produced by DAB and the H_2_O_2_ levels in rice leaves were recorded at 1 and 3 dpi with PXO99^A^, Δ*xanA*, Δ*imp* or H_2_O. As shown in Fig. 3, H_2_O-inoculated leaves served as the blank control, and a slight oxidative burst could be detected around the injection sites due to the wound inoculation method. When compared with PXO99^A^-inoculated leaves at 1 and 3 dpi, both Δ*xanA-* and Δ*imp*-inoculated leaves showed much darker orange-brown deposits and significantly higher H_2_O_2_ levels. Notably, Δ*imp*-inoculated leaves generated a relatively stronger oxidative burst than Δ*xanA*-inoculated leaves at 1 dpi, while at 3 dpi, Δ*imp*-inoculated leaves were found to have relatively weaker oxidative bursts than Δ*xanA*-inoculated leaves. These results indicated that the reduced virulence of ∆*xanA* and ∆*imp* might be associated with a dramatic difference in elicitation of rice defense responses.

### Identification and Functional Classification of DEGs in Rice Inoculated with PXO99^A^ Relative to those in Rice Inoculated with the Mutants ∆*xanA* and ∆*imp*

**Fig. 4:**
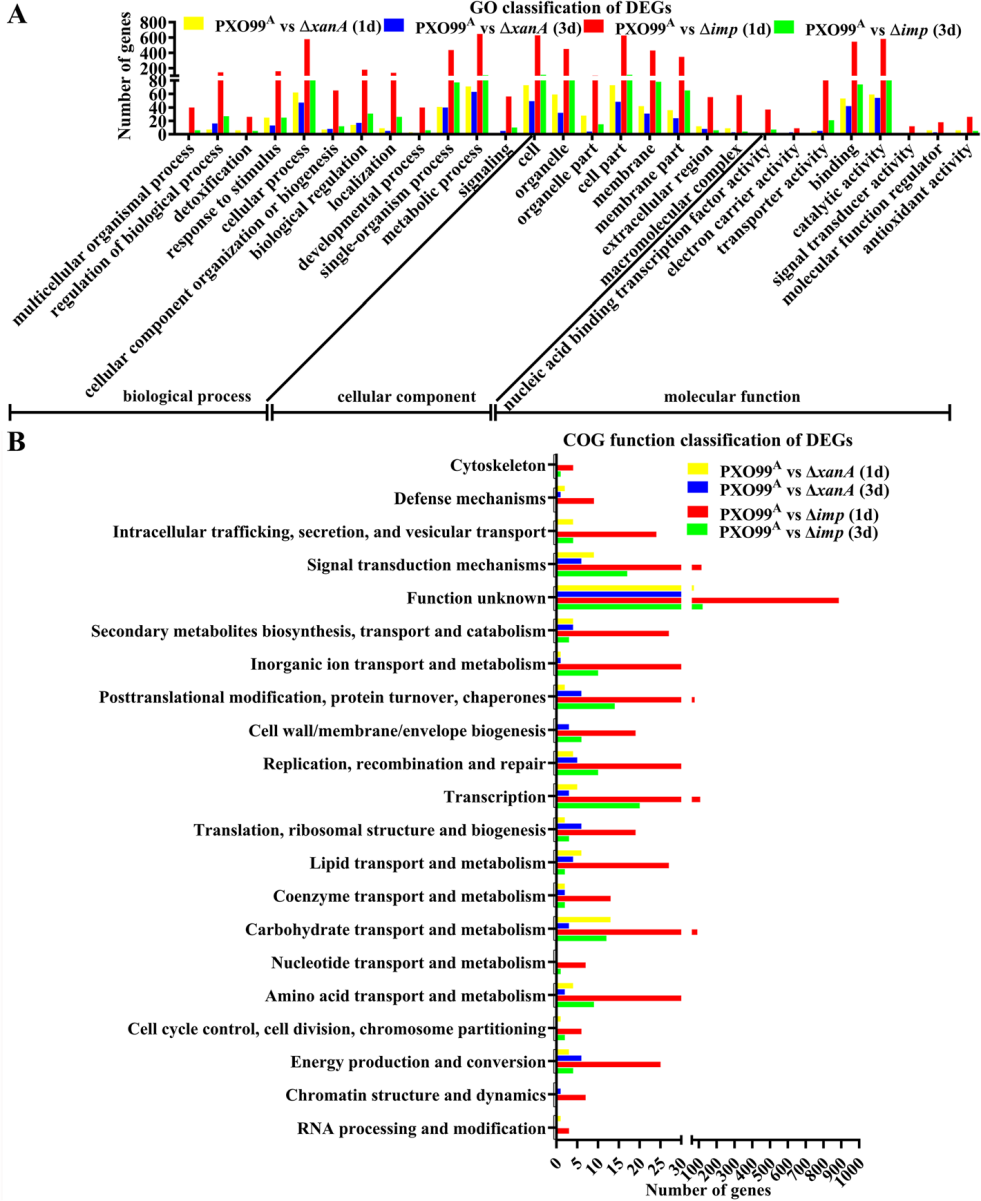
Histogram presentation of Gene Ontology (GO) and Clusters of Orthologous Groups (COGs) classifications of DEGs from four pairwise comparisons: PXO99^A^ vs Δ*xanA* (1d), PXO99^A^ vs Δ*xanA* (3d), PXO99^A^ vs Δ*imp* (1d) and PXO99^A^ vs Δ*imp* (3d). **A** GO annotations and classifications of the DEGs. All DEGs from each pairwise comparison were classified into three main GO categories (biological process, cellular component and molecular function) and 28 dominant subcategories were presented. The x-axis shows the names of GO terms and categories. The y-axis indicates the number of DEGs in each category. **B** COG annotations and classifications of the DEGs. All DEGs from each pairwise comparison were assigned to 21 categories in the COG classification. The y-axis shows the description of the 21 functional categories, and the x-axis indicates the number of DEGs in each category. Any one DEG may be categorized into different GO and COG classes. Detailed information is shown in Table S2 and Table S3

To compare gene expression changes in Δ*xanA*-, Δ*imp*- and PXO99^A^-treated rice leaves at 1 and 3 dpi, we analyzed the transcriptome profiles of PXO99^A^_1d, PXO99^A^_3d, ∆*imp*_1d, ∆*imp*_3d, ∆*xanA*_1d and ∆*xanA*_3d samples. The statistical summary of the transcriptome data is presented in Table 2. The relative expression levels were calculated by using the PXO99^A^ treatment as the control, and the results revealed that 1521 (512 upregulated; 1009 downregulated), 227 (51 upregulated; 176 downregulated), 131 (39 upregulated; 92 downregulated) and 106 (75 upregulated; 31 downregulated) genes were identified as DEGs in the comparison groups PXO99^A^ vs Δ*imp* (1d), PXO99^A^ vs Δ*imp* (3d), PXO99^A^ vs Δ*xanA* (1d) and PXO99^A^ vs Δ*xanA* (3d), respectively (log_2_|fold changes| > 1 and *p* value < 0.05; Table 2; Table S1). The Gene Ontology (GO) and Clusters of Orthologous Groups (COGs) databases were used to further classify the potential functions of the DEGs. As shown in Fig. 4A and Table S2, the annotated DEGs from all comparison groups were classified into three main GO categories (biological process, cellular component and molecular function) and 28 dominant subcategories were presented. Among them, the GO terms metabolic process, cellular process, single-organism process, response to stimulus, cell, organelle, membrane, catalytic activity, binding and antioxidant activity were dominant in each of the four comparison groups (Fig. 4A). However, the number of annotated genes between comparison groups differed. According to COG annotation, the annotated DEGs from all comparison groups could be classified into 21 different functional categories (Fig. 4B, Table S3). The results showed that the known functional categories of the dominant DEGs were involved in K (transcription), T (signal transduction mechanisms), O (posttranslational modification, protein turnover and chaperones), G (carbohydrate transport and metabolism) and L (replication, recombination and repair) both in the comparison groups PXO99^A^ vs Δ*imp* (1d) and PXO99^A^ vs Δ*imp* (3 dpi). The dominant functional categories in the comparison group PXO99^A^ vs Δ*xanA* (1 dpi) were G (carbohydrate transport and metabolism), T (signal transduction mechanisms) I (lipid transport and metabolism) and K (transcription). However, in the comparison group PXO99^A^ vs Δ*xanA* (3 dpi), the dominant functional categories were C (energy production and conversion), J (translation, ribosomal structure and biogenesis), O (posttranslational modification, protein turnover, chaperones) and T (signal transduction mechanisms). Interestingly, there were 9 and 2 DEGs involved in V (defense mechanisms) in groups PXO99^A^ vs Δ*imp* (1 dpi) and PXO99^A^ vs Δ*xanA* (1 dpi), respectively. In comparison, the data of groups PXO99^A^ vs Δ*imp* (3d) and PXO99^A^ vs Δ*xanA* (3 dpi) were as low as 0 and 1, respectively.

**Table 2:** Summary of transcriptome sequencing data produced by Illumina sequencing

Sample	Raw reads	Clean reads	Q20(%)^**a**^	Total mapped	Multiple mapped	Uniquely mapped	Comparison groups	Total DEGs^**b**^	DEGs (↑) ^**b**^	DEGs (↓) ^**b**^
PXO99^A^_1d1	41,010,840	38,918,214	97.37	36,505,039 (93.8%)	1,810,186 (4.65%)	34,694,853 (89.15%)	PXO99^A^ vs Δ*imp* (1d)			
PXO99^A^_1d2	43,382,564	40,471,568	98.04	38,005,434 (93.91%)	1,189,639 (2.94%)	36,815,795 (90.97%)		1521	512	1009
PXO99^A^_1d3	46,857,330	43,599,626	97.43	40,981,207 (93.99%)	1,400,984 (3.21%)	39,580,223 (90.78%)				
PXO99^A^_3d1	41,322,886	38,515,876	98.02	36,206,414 (94.0%)	1,032,570 (2.68%)	35,173,844 (91.32%)				
PXO99^A^_3d2	42,797,162	40,443,546	97.68	38,023,873 (94.02%)	1,327,664 (3.28%)	36,696,209 (90.73%)				
PXO99^A^_3d3	41,168,536	39,233,110	97.63	36,695,566 (93.53%)	1,663,516 (4.24%)	35,032,050 (89.29%)	PXO99^A^ vs Δ*imp* (3d)			
imp_1d1	43,654,506	42,143,374	98.27	39,671,695 (94.14%)	1,815,819 (4.31%)	37,855,876 (89.83%)		227	51	176
imp_1d2	42,779,594	42,036,494	98.2	39,606,885 (94.22%)	1,597,159 (3.8%)	38,009,726 (90.42%)				
imp_1d3	44,399,584	43,569,548	98.24	40,818,028 (93.68%)	2,022,181 (4.64%)	38,795,847 (89.04%)				
imp_3d1	46,118,502	45,285,946	98.22	42,528,243 (93.91%)	1,860,138 (4.11%)	40,668,105 (89.8%)				
imp_3d2	45,012,320	40,910,822	98.06	38,467,718 (94.03%)	1,574,795 (3.85%)	36,892,923 (90.18%)	PXO99^A^ vs Δ*xanA* (1d)			
imp_3d3	46,332,702	43,541,914	98.21	40,911,915 (93.96%)	1,550,638 (3.56%)	39,361,277 (90.4%)		131	39	92
xanA_1d1	44,739,304	41,884,126	97.65	39,206,960 (93.61%)	1,336,065 (3.19%)	37,870,895 (90.42%)				
xanA_1d2	43,063,064	40,742,626	97.65	38,212,980 (93.79%)	1,217,254 (2.99%)	36,995,726 (90.8%)				
xanA_1d3	41,941,700	41,167,914	98.21	38,935,614 (94.58%)	1,732,387 (4.21%)	37,203,227 (90.37%)				
xanA_3d1	45,891,720	45,053,084	98.25	42,570,896 (94.49%)	2,120,406 (4.71%)	40,450,490 (89.78%)	PXO99^A^ vs Δ*xanA* (3d)			
xanA_3d2	44,893,464	44,085,224	98.27	41,603,500 (94.37%)	2,577,904 (5.85%)	39,025,596 (88.52%)		106	75	31
xanA_3d3	43,236,992	42,496,768	98.26	39,661,084 (93.33%)	1,542,173 (3.63%)	38,118,911 (89.7%)				

^a^Q20(%): The proportion of nucleotides with quality value larger than 20 in reads

^b^DEGs: Differentially expressed genes. ↑: Upregulated. ↓: Downregulated

### DEG Enrichment Analyses Based on the GO and KEGG Databases

**Fig. 5:**
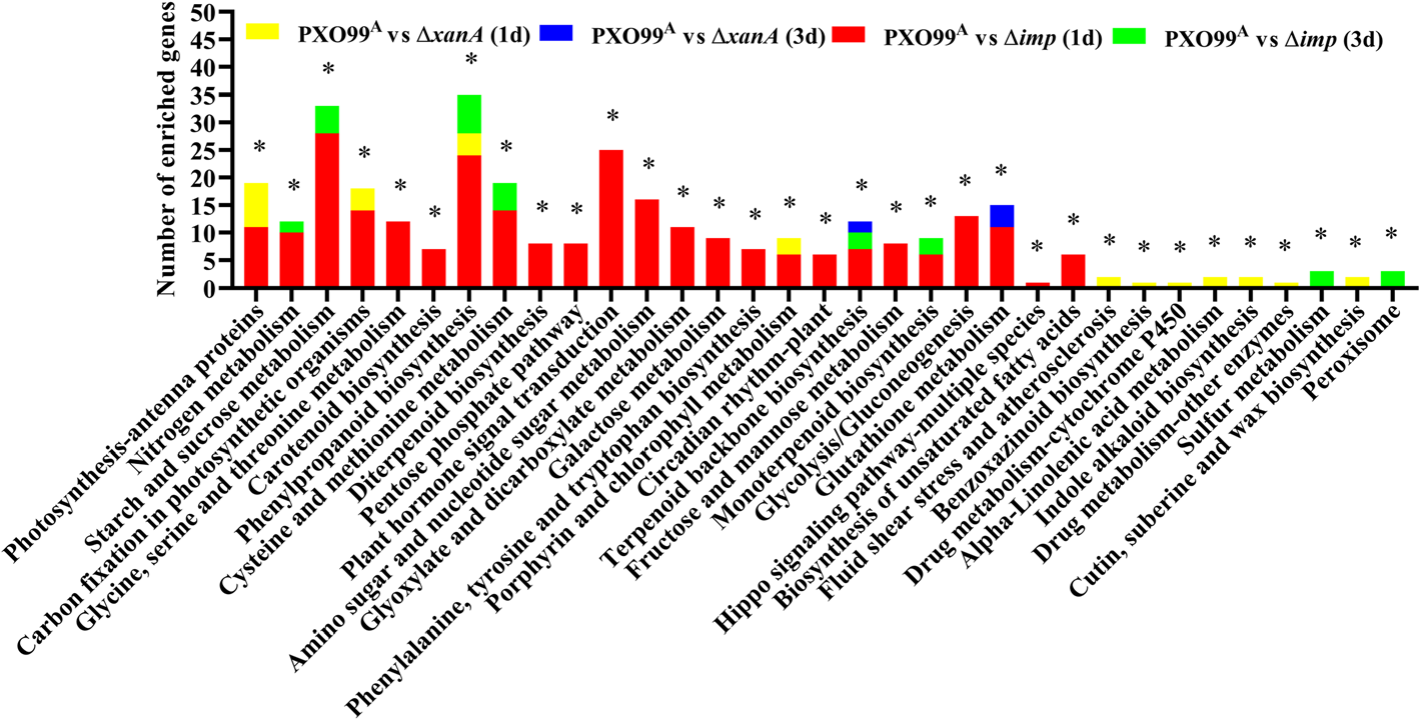
KEGG pathway enrichment analysis of DEGs from comparison groups PXO99^A^ vs Δ*xanA* (1d), PXO99^A^ vs Δ*xanA* (3d), PXO99^A^ vs Δ*imp* (1d) and PXO99^A^ vs Δ*imp* (3d). Histogram of the top 33 significantly enriched pathways with the highest representation of the DEGs. The names of the KEGG pathways are listed along the x-axis. The y-axis indicates the number of enriched genes in different comparison groups. Asterisks indicate significant enrichment (*P < 0.05). The detailed information is shown in Table S4

We also performed enrichment analyses of DEGs based on the GO and KEGG databases to further identify some significantly enriched pathways related to the Δ*xanA* and Δ*imp* treatments at the early stages of infection. As shown in Fig. 5 and Table S4, KEGG pathway enrichment analysis (*p* value ≤0.05) at 1 dpi revealed that DEGs related to “Photosynthesis-antenna proteins”, “Carbon fixation in photosynthetic organisms”, “Phenylpropanoid biosynthesis” and “Porphyrin and chlorophyll metabolism” were enriched both in groups PXO99^A^ vs Δ*imp* (1d) and PXO99^A^ vs Δ*xanA* (1d). Furthermore, “Nitrogen metabolism”, “Starch and sucrose metabolism”, “Glycine, serine and threonine metabolism”, “Carotenoid biosynthesis”, “Cysteine and methionine metabolism” and “Plant hormone signal transduction” were major pathways specifically enriched in group PXO99^A^ vs Δ*imp* (1d) at 1 dpi, while “Fluid shear stress and atherosclerosis”, “Benzoxazinoid biosynthesis”, “Drug metabolism-cytochrome P450”, “alpha-Linolenic acid metabolism” and “Indole alkaloid biosynthesis” were major pathways specifically enriched in group PXO99^A^ vs Δ*xanA* (1d) at 1 dpi. When compared at 3 dpi, only DEGs related to “Terpenoid backbone biosynthesis” were enriched both in groups PXO99^A^ vs Δ*imp* (3d) and PXO99^A^ vs Δ*xanA* (3d), and another pathway “Glutathione metabolism” was specifically enriched in group PXO99^A^ vs Δ*xanA* (3d). Notably, “Nitrogen metabolism”, “Starch and sucrose metabolism”, “Phenylpropanoid biosynthesis”, “Cysteine and methionine metabolism”, “Terpenoid backbone biosynthesis” and “Monoterpenoid biosynthesis” were continuously enriched at 1d and 3d after Δ*imp* treatment. Additionally, as shown in Figure S2 and Table S5, GO enrichment analysis (FDR ≤ 0.05) revealed that the annotated DEGs from different comparison groups were summarized into three main GO categories: biological process, molecular function, and cellular component. The top 5 significantly enriched GO terms (“Photosynthesis, light harvesting in photosystem i”, “Photosynthesis, light harvesting”, “Generation of precursor metabolites and energy”, “Response to light stimulus” and “Oxidation-reduction process”) in biological process, the top 5 significantly enriched GO terms (“Pigment binding”, “Chlorophyll binding”, “Tetrapyrrole binding”, “Oxidoreductase activity, acting on peroxide as acceptor” and “Oxidoreductase activity”) in molecular function and the top 5 significantly enriched GO terms (“Photosystem”, “Photosystem i”, “Plastoglobule”, “Plastid part” and “Chloroplast part”) in cellular component were identified at 1 dpi both in groups PXO99^A^ vs Δ*imp* (1d) and PXO99^A^ vs Δ*xanA* (1d). Furthermore, “Carbohydrate transport”, “Carbohydrate metabolic process”, “Carbohydrate transmembrane transport”, “Detoxification”, “Heme binding” and “Peroxidase activity” were major GO terms specifically enriched in group PXO99^A^ vs Δ*imp* (1d), while “Response to stimulus”, “Thylakoid part”, “Envelope”, “Thylakoid membrane” and “Photosynthetic membrane” were major GO terms specifically enriched in group PXO99^A^ vs Δ*xanA* (1d). Unlike at 1 dpi, only two GO terms, “oxidoreductase activity, oxidizing metal ions, NAD or NADP as acceptor” and “ion transport”, were found to be significantly enriched in group PXO99^A^ vs Δ*imp* (3 d) at 3 dpi and none of the GO terms were significantly enriched in group PXO99^A^ vs Δ*xanA* (3d) at 3 dpi. Taken together, these results indicated that *xanA* and *imp* may play important roles in affecting photosynthesis, metabolism processes and biotic stress response of rice at early stages of *Xoo* infection.

### Overview of the Roles of Host-Induced Virulence Factors (XanA and Imp) of *Xoo* in the Metabolic Processes of Rice

**Fig. 6:**
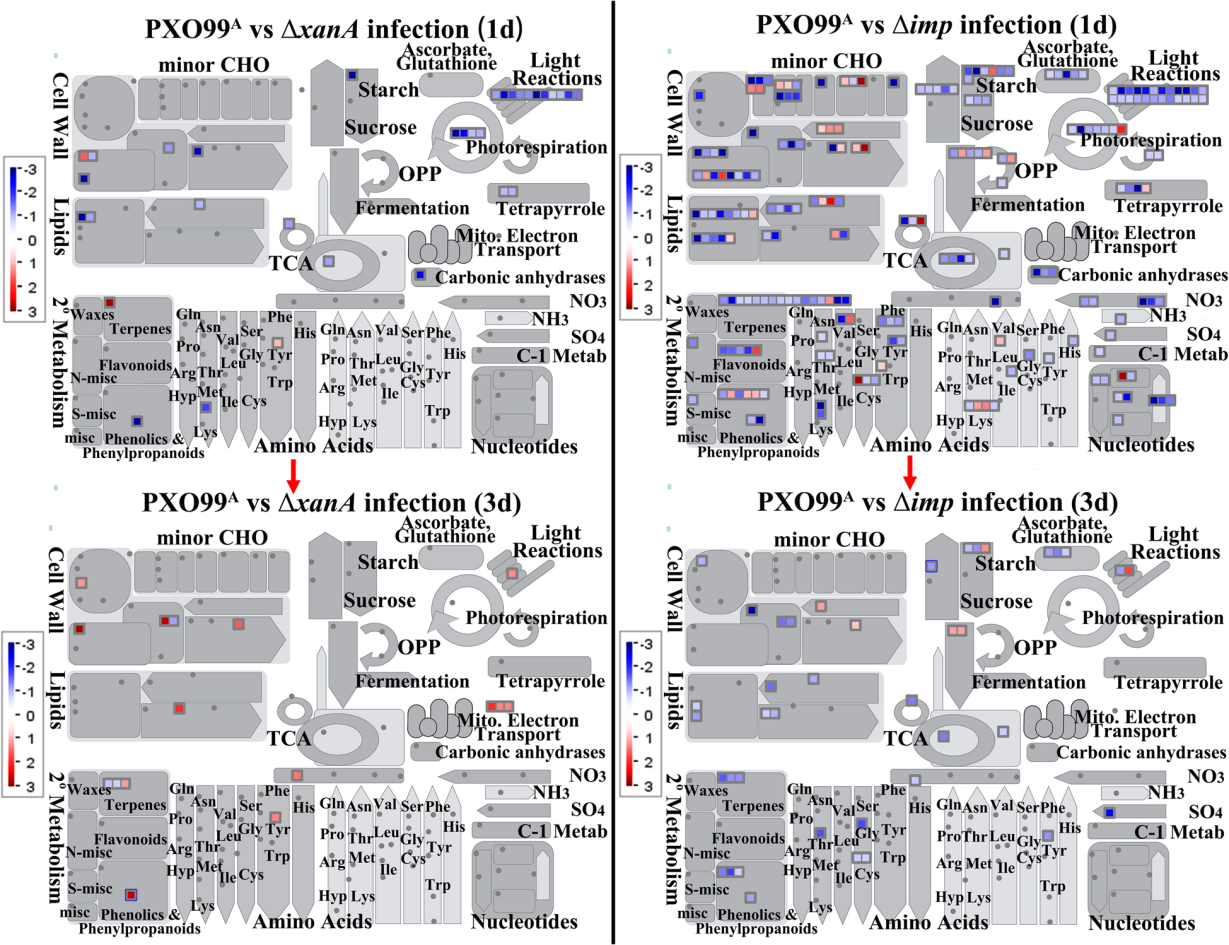
MapMan analysis and comparison of the metabolic changes in IR24 rice at 1 and 3 d after infection with the mutants Δ*xanA* and Δ*imp* relative to those with PXO99^A^ infection. In each comparison group, the corresponding DEGs with |log2 (fold change)| ≥ 1 were imported into MapMan software. The gray circles indicate that no differentially expressed genes were matched in this process. The red and blue squares attached to each metabolic pathway represent up- and downregulated genes, respectively. The color intensity represents the gene expression level (log2 ratio mutant/PXO99^A^), as indicated by the color scale. Detailed information is shown in Table S6

To better clarify the biological roles of host-induced virulence factors (XanA and Imp), a comparison and overview of the metabolic processes in rice transcriptionally affected by PXO99^A^, Δ*imp* and Δ*xanA* infection was obtained by uploading the gene expression profiles of different comparison groups to the MapMan toolkit. As shown in Fig. 6 and Table S6, when compared with PXO99^A^-treated rice at 1 dpi, both of Δ*xanA*-treated rice and Δ*imp*-treated rice showed significant down regulation of most genes involved in photosynthesis (Calvin cycle and light reaction), cell wall metabolism, tricarboxylic acid (TCA) cycle, lipid metabolism, major carbohydrate metabolism, tetrapyrrole synthesis, secondary metabolism (simple phenols) and amino acid metabolism (homoserine), indicating that both XanA and Imp of *Xoo* could promote photosynthesis and metabolic processes of rice at early infection stage. Additionally, several rice genes involved in flavonoids, amino acid degradation, minor carbohydrate metabolism, glycolysis, OPP (the oxidative and nonreductive pentose phosphate pathway), redox (ascorbate and glutathione), nucleotide metabolism, N metabolism and S assimilation showed significant expression differences only in group PXO99^A^ vs Δ*imp* (1d). Unlike 1dpi, when compared with PXO99^A^-treated rice at 3 dpi, the gene expression patterns of Δ*xanA*-treated rice and Δ*imp*-treated rice are quite different, and there are little overlapped MapMan pathway (Fig. 6 and Table S6). For example, almost all genes related to photosynthesis, mitochondrial electron transport, amino acid metabolism, lipid metabolism and cell wall metabolism were significantly upregulated at 3dpi after Δ*xanA* treatment, while numerous genes related to secondary metabolism, major carbohydrate metabolism, lipid metabolism, cell wall metabolism, amino acid metabolism, TCA cycle, redox (ascorbate and glutathione) and S-assimilation were significantly repressed by Δ*imp* treatment at 3dpi. We suspect that XanA and Imp might exploite different working pathways to help *Xoo* modify rice metabolic processes. Taken together, these results reveal that *Xoo* infection has a significant impact on rice metabolism and that a proportion can be attributed to the presence of host-induced virulence factors XanA and Imp.

### Rice DEGs Associated with the Biotic Stress Pathway in Response to Treatment with the Mutants ∆*xanA* and ∆*imp* Relative to those in Response to PXO99^A^

**Fig. 7:**
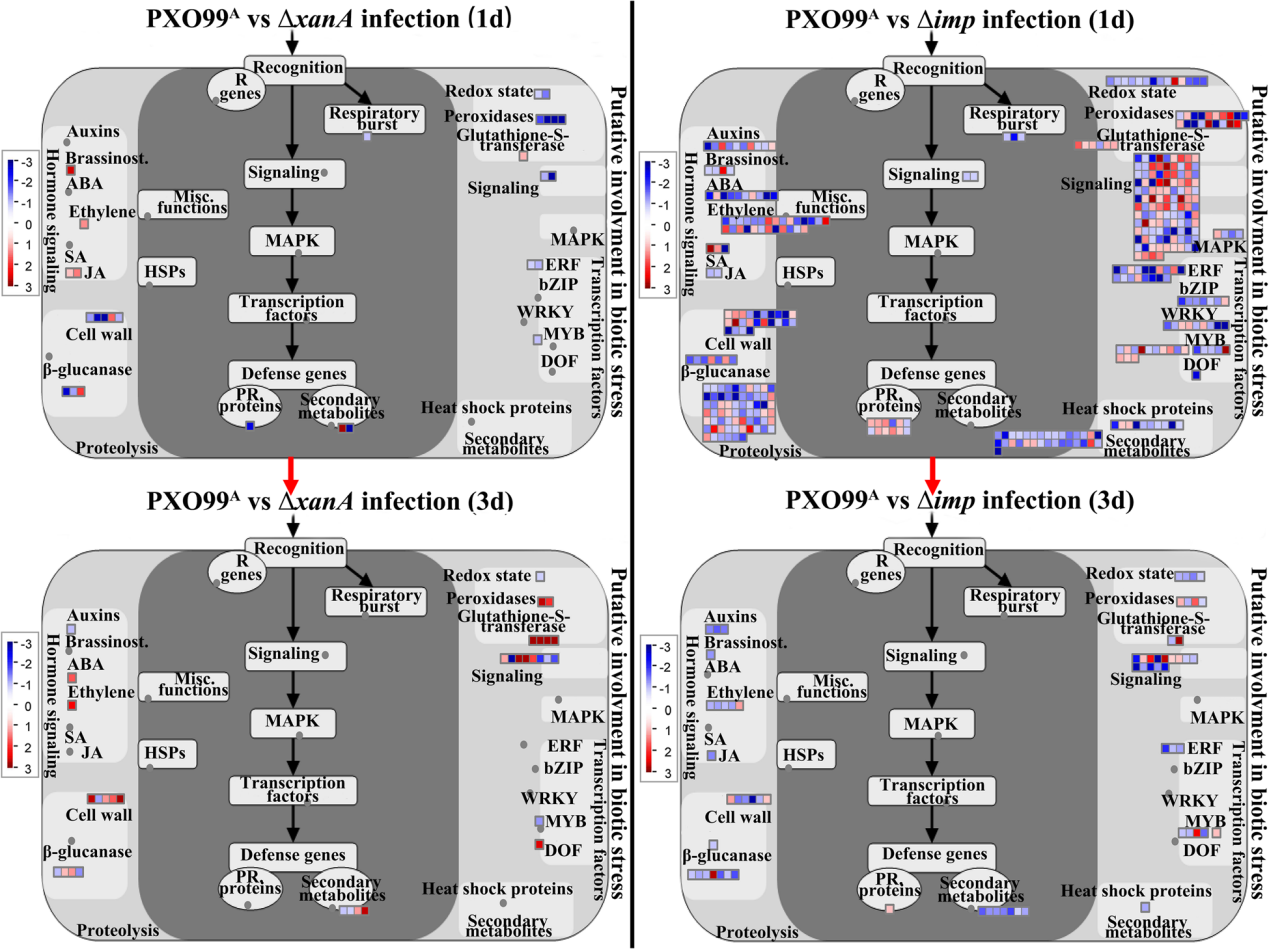
MapMan visualization and comparison of the DEGs associated with the biotic stress pathway among IR24 rice infected by PXO99^A^, Δ*xanA* or Δ*imp* at 1 and 3 dpi. In each comparison group, the corresponding DEGs with |log2 (fold change)| ≥ 1 were imported into MapMan software. The gray circles indicate missing data. The DEGs successfully matched to the biotic stress pathway are represented by colored squares, where the red squares and blue squares indicate up- and downregulated genes, respectively. The color intensity represents the gene expression level (log2 ratio mutant/PXO99^A^), as indicated by the color scale. Detailed information is presented in Table S7

To acquire further insights into the role of the host-induced virulence factors XanA and Imp in the rice biotic stress response, the MapMan toolkit was used to map the DEGs of different comparison groups to pathways involved in plant-pathogen interactions. As shown in Fig. 7 and Table S7, the DEGs from four comparison groups with known functions such as R genes, signaling, MAPK cascades, respiratory burst, TFs, PRs, heat shock proteins, peroxidases, etc. were identified. Notably, the Δ*xanA*-treated rice exhibit continuous up-regulation of genes involved in hormone signaling and glutathione S transferase both at 1 dpi and 3dpi. For Δ*imp*-treated rice at 1 dpi and 3dpi, most of hormone signaling related genes and heat shock proteins related genes were continuously downregulated and most of PRs related genes were continuously upregulated. These results suggest that pathways such as hormone signaling, glutathione S transferase, heat shock proteins and PRs may be important for rice to resist infection of Δ*imp* and Δ*xanA*. When compared with PXO99^A^-treated rice at 1 dpi, most of genes involved in hormone signaling of auxins, brassinosteriod, ABA, JA and ethylene were downregulated in Δ*imp*-treated rice, whereas all genes involved in hormone signaling of brassinosteriod, JA and ethylene were upregulated in Δ*xanA*-treated rice. Additionally, all genes involved in PRs, respiratory burst, redox state, peroxidases, TFs (ERF), TFs (MYB) and signalling were downregulated in Δ*xanA*-treated rice at 1dpi. However, except for all respiratory burst related genes that were downregulated, the genes involved in above MapMan pathways were either upregulated or downregulated in Δ*imp*-treated rice. When compared with PXO99^A^-treated rice at 3 dpi, most of genes associated with signaling, hormone signaling of ABA and ethylene, glutathione S transferases, peroxidases and TFs (DOF) were upregulated in Δ*xanA*-treated rice at 3 dpi, while in Δ*imp*-treated rice, most of genes associated with signalling, hormone signaling of auxins, brassinosteriod, ethylene and JA, redox state, TFs (ERF), TFs (MYB) and heat shock proteins were downregulated. (Fig. 7 and Table S7). These results indicated that the host-induced virulence factors XanA and Imp might have key roles in the rice biotic stress response, and all of the DEGs mapped in the overviews will be useful targets for understanding the molecular mechanisms involved in XanA and Imp at the early stage of *Xoo* infection.

### Validation of DEGs from RNA-Seq Data Using qRT-PCR

**Fig. 8:**
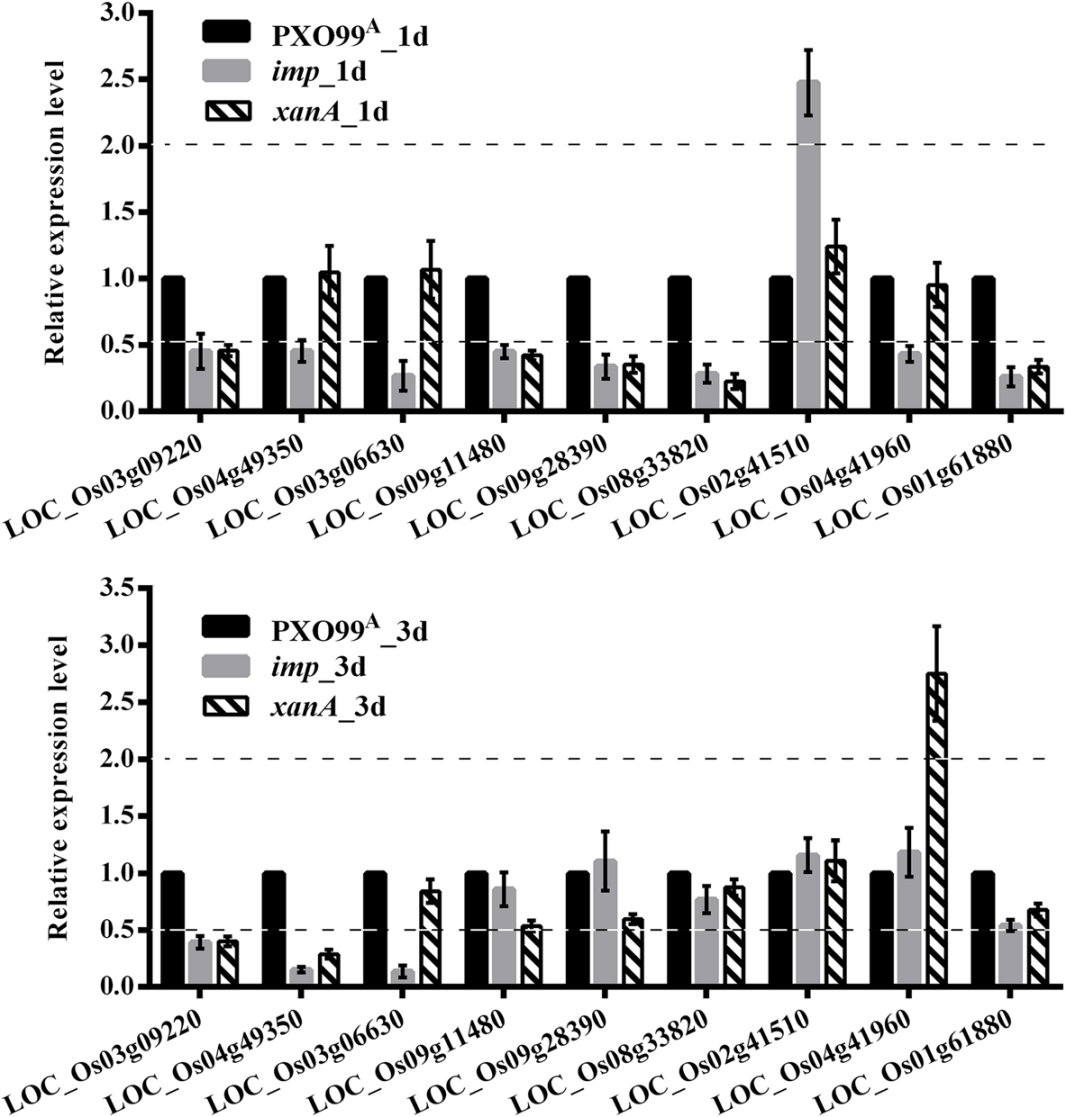
The qRT-PCR validation of 9 randomly selected DEGs from RNA-Seq data. The genes *eEF1a* and *actin* were used as internal standard. PXO99^A^_1d and PXO99^A^_3d were as control groups at 1dpi and 3dpi respectively. Gene expression level in the control group was set to 1.0. The data were expressed as the mean fold change (means ± SD, *n* = 3) relative to the corresponding control group. The values were significantly different to the control when the relative expression level (fold change) ≥ 2 or ≤ 0.5

To verify that the DEGs identified by RNA-seq were indeed differentially expressed, a total of nine genes were randomly selected for validation using qRT-PCR. As shown in Fig. 8, LOC_Os03g09220 (BTH-induced protein phosphatase 2C 1) was significantly downregulated in samples *imp*_1d, *imp*_3d, *xanA*_1d and *xanA*_3d compared with their controls. LOC_Os04g49350 (pentatricopeptide repeat domain containing protein) showed significantly lower expression levels in samples *imp*_1d, *imp*_3d and *xanA*_3d than in their controls. Both in samples *imp*_1d and *imp*_3d, LOC_Os03g06630 (heat stress transcription factor) was significantly downregulated compared to the control levels. Significantly lower expression levels of LOC_Os09g11480 (ethylene-responsive transcription factor), LOC_Os09g28390 (abscisic acid 8′-hydroxylase 3), LOC_Os08g33820 (chlorophyll A-B binding protein) and LOC_Os01g61880 (respiratory burst oxidase) were all detected in samples *imp*_1d and *xanA*_1d than in control samples. In addition, LOC_Os02g41510 (R2R3-type MYB transcription factor) showed significantly higher expression levels in sample *imp*_1d than in PXO99^A^_1d. When compared to control samples, LOC_Os04g41960 (NADP-dependent oxidoreductase) was significantly downregulated in sample *imp*_1d and was significantly upregulated in sample *xanA*_3d. These qRT-PCR analyses showed that the expression patterns of the chosen genes were consistent with those shown by the RNA-Seq data (Fig. 8, Table S1), although there were some differences in the degree of the changes. These results indicated that the RNA-Seq results were reliable.

## Discussion

Despite the significant advances in the understanding of interactions of *Xanthomonas* species with their host plants in past decades, many bacterial determinants induced by hosts that contribute to *Xanthomonas* virulence and account for alterations in host response and gene expression have yet to be identified. One of the important findings of the present study is that the in vitro interaction system combined with comparative proteomics analyses revealed 4 virulence-related proteins that were continuously induced by the host at 3, 6 and 12 h after rice leaf extract (RLX) treatment (Fig. 1 and Table 1). Among them, the septum site-determining protein (MinD), a member of the bacterial Min-system, is involved in regulating septum site selection and cell division events in many Gram-negative organisms (England et al. 2011). Here, we identified MinD as a novel virulence determinant in *Xoo*, and RLX treatment simultaneously induced downregulated expression of the MinD protein. We noticed that RLX treatment simultaneously induced upregulated expression of arginase RocF, which we identified as a virulence determinant in *Xoo*. Arginase (RocF) is a key enzyme in the urea cycle, hydrolyzing L-arginine to L-ornithine and urea in both bacteria and eukaryotes. Zhang et al. also revealed that the protein expression level of RocF can be induced by rice and arginine, and deletion of the *rocF* gene significantly attenuated the virulence of *Xoo* in rice (Zhang et al. 2019), which can be regarded as evidence supporting the reliability of our results. Notably, RLX treatment also simultaneously induced the upregulated expression of two carbon metabolism-related enzymes, inositol monophosphatase (Imp) and phosphohexose mutase (XanA). Inositol monophosphatase primarily dephosphorylates inositol monophosphate to maintain the cellular inositol pool, which is crucial in the phosphatidylinositol (PI) signaling pathway, and SuhB, a homolog of inositol monophosphatase, has been demonstrated to be induced during the interaction between *Pseudomonas aeruginosa* and its host (Li et al. 2013). Phosphohexose mutase (XanA) catalyzes phosphoryl transfer between hexose-6-phosphate and hexose-1-phosphate and plays important roles in carbon metabolism and polysaccharide synthesis in many organisms (Goto et al. 2016). Notably, we demonstrated herein, for the first time, that both mutants ∆*xanA* and ∆*imp* in *X. oryzae* pv. *oryzae* almost completely failed to stimulate disease symptoms, which is one of the main reasons why we chose to focus on investigating the effects of these two carbohydrate metabolism-related enzymes on the rice response and gene expression.

Clearly, the leaves of the susceptible rice IR24 exhibited two different types of symptoms after infection with PXO99^A^ and the two avirulent mutants (∆*xanA* and ∆*imp*) (Fig. 2). PXO99^A^-treated rice leaves exhibited long chlorotic lesions because *Xoo*, like many other plant pathogenic bacteria, deploys a diverse set of virulence strategies to overcome or attenuate the defense responses of susceptible hosts and establish a favorable niche for bacterial growth (Yang et al. 2005, Hersemann et al. 2017). However, the two avirulent mutants, without the host-induced carbohydrate metabolism enzymes XanA and Imp, were countered by the immune response of the susceptible rice, and infection was inhibited. The oxidative burst, a rapid accumulation of reactive oxygen species (ROS), is an early and complex defense reaction induced by biotic stresses and plays diverse roles in plant-pathogen interactions (Wang et al. 2019). In this study, we examined the generation of oxidative bursts and the levels of ROS in an attempt to determine the roles of two host-induced metabolic enzymes, XanA and Imp, in the resistance reactions of susceptible rice to *Xoo*. The results showed that PXO99^A^, ∆*xanA* and ∆*imp* induced oxidative bursts in susceptible rice at 1 and 3 dpi, and the intensity of the oxidative burst and levels of ROS, mostly H_2_O_2_, both in ∆*xanA*- and ∆*imp*-treated rice leaves were significantly higher than those in PXO99^A^-treated rice leaves during the entire study period (Fig. 3); however, the molecular mechanisms underlying the enhanced resistance of susceptible rice to ∆*xanA*- and ∆*imp* might be different. Regarding Imp, loss of SuhB, an Imp homolog in *Pseudomonas aeruginosa*, resulted in avirulence and suppression of T3SS gene expression (Li et al. 2013). Therefore, we speculate that the suppression of T3SS and its effectors in ∆*imp* leads to the fact that ∆*imp* infection cannot overcome the PAMP-triggered immunity (PTI) of the susceptible rice and the consequently enhanced defense response. Regarding XanA, it has been confirmed that XanA is highly conserved in *Xanthomonas* spp. and is required for the synthesis of Xanthan, a kind of extracellular polysaccharose (EPS) (Musa et al. 2013). A recent study suggested that EPS might act as a suppressor of PTI during *X. oryzae* pv. *oryzae* infection of rice, and pretreatment of rice leaves with an EPS-deficient mutant significantly elicited a rice immune response to subsequent infection by the wild-type strain (Girija et al. 2017). These results suggest the possibility that the EPS-deficient mutant ∆*xanA* is defective in the suppression of rice immunity and is an enhancer of rice defense responses.

In this study, two avirulent mutants of *Xoo*, ∆*xanA* and ∆*imp*, were used to study the DEGs involved in rice global responses underlying the important roles of host-induced carbohydrate metabolism enzymes in the *Xoo*-rice interaction. We identified 1521 and 131 DEGs in the comparison groups PXO99^A^ vs ∆*imp* (1 d) and PXO99^A^ vs ∆*xanA* (1 d), respectively at the early infection stage (1 dpi). Interestingly, the number of DEGs decreased to 227 in group PXO99^A^ vs ∆*imp* (3 d) and to 106 in group PXO99^A^ vs ∆*xanA* (3d) at 3 dpi (Fig. 4 and Table 2), indicating that a counterbalance between a timely response mechanism and the gradual adaptation mechanism existed in rice following treatment with the two avirulent mutants compared to the PXO99^A^ treatment. The time-resolved RNA-Seq analysis of the susceptible rice JG30 inoculated with PXO99^A^ and the avirulent mutant PH also showed a similar phenomenon in which the number of DEGs peaked at 24 hpi and decreased gradually thereafter (Tariq et al. 2019). Furthermore, a comparative transcriptomic study of the resistant rice genotype CBB23 and the susceptible rice genotype JG30 during different stages of PXO99^A^ infection revealed that the number of DEGs peaked at 12 hpi and decreased steadily thereafter (Tariq et al. 2018). Using Venn diagram analysis and functional annotation, we revealed a total of 116 overlapping DEGs that responded to both ∆*xanA* and ∆*imp* treatments at 1 and 3 dpi and found that these overlapping DEGs were mainly involved in carbohydrate metabolism, photosynthesis, signal transduction, secondary metabolism, amino acid metabolism, transcription and posttranslational modification (Figure S3 and Table S8). Interestingly, Venn diagram analysis showed that 83 genes implicated in signal transduction, transcription, carbohydrate metabolism, posttranslational modification, secondary metabolism and ion transport were continuously differentially expressed both at 1 and 3 d after ∆*imp* treatment, while only two genes associated with carbohydrate metabolism and signal transduction were continuously differentially expressed in response to ∆*xanA* treatments at 1 and 3 dpi (Figure S3 and Table S8). The above results indicate that rice recognition and response events related to timely counteraction of different biotic stresses most likely occurred at the early infection stage. Although the ∆*xanA* and ∆*imp* treatments caused similar avirulent phenotypes and enhanced defense responses, the molecular basis of the rice regulatory mechanisms were not exactly the same.

Molecular antioxidants, such as ascorbate and glutathione, are required for the detoxification of ROS-mediated damage in plants (Kangasjarvi et al. 2008). In this study, we observed that genes related to ascorbate and glutathione were specifically downregulated both at 1 and 3 dpi after ∆*imp* infection (Fig. 6 and Table S6), which might be one of the reasons for the relatively high levels of ROS in ∆imp-treated tissues. In addition, antioxidative enzymes, including glutathione-S-transferases, thioredoxin and peroxidases, contribute to the cellular redox balance by scavenging ROS and play important roles in abiotic and biotic stress modulation pathways of plants (Das and Roychoudhury 2014). Here, the expression of peroxidase-related genes in ∆*xanA-*treated rice leaves was downregulated at 1 dpi and then upregulated at 3 dpi; however, the peroxidase-related genes in ∆*imp-*treated rice leaves showed varied expression patterns at 1 dpi and 3 dpi (Fig. 6 and Table S6). Furthermore, early studies on the role of GSTs in plant biotic stress showed that certain GST genes are markedly induced in the early phase of microbial infections (Gullner et al. 2018). Our study showed that the vast majority of genes related to glutathione S transferases exhibited upregulated expression after ∆*imp* and ∆*xanA* infection compared with that after PXO99^A^ infection both at 1 and 3 dpi. These transcriptome-wide investigations not only reveal the diverse roles of XanA and Imp in affecting ROS-related gene expression but also provide novel insight into the mechanisms underlying the enhanced ROS levels in ∆*xanA*- and ∆*imp*-treated rice leaves.

Leaf photosynthesis plays important roles in determining crop yield, and previous studies indicate that plants reduce the expression of photosynthesis-related genes during biotic stress as a defense strategy, likely for energy conservation and restriction of the availability of nutrient sources for pathogens (Bilgin et al. 2010, Yu et al. 2014). Moreover, it was found in rice that genes involved in photosynthetic functions were repressed after PXO99^A^ infection or bacterial PAMP (LPS) treatment (Narsai et al. 2013, Girija et al. 2017). In the current study, GO, KEGG and MapMan analyses revealed that most DEGs related to photosynthesis, including light reactions, the Calvin cycle and tetrapyrrole, were significantly enriched and were downregulated after ∆*xanA* and ∆*imp* infection compared with that after PXO99^A^ infection at early stages (1 dpi) (Fig. 6, Figure S4 and Table S6). These results suggest that the lack of *xanA* or *imp* in the mutant strains might trigger higher suppression of photosynthesis and stronger rice defense responses than PXO99^A^. Lipid metabolism is also thought to provide important sources of reserve energy, which is particularly crucial for the energy-intensive processes that underlie the plant defense response to pathogen infection (Lim et al. 2017). Here, KEGG and MapMan analysis revealed that most DEGs enriched in the lipid metabolism pathway were significantly repressed in ∆*imp* relative to PXO99^A^-treated rice both at 1 dpi and 3 dpi. Although the lipid metabolism-related genes were also downregulated in ∆*xanA* relative to those in PXO99^A^-treated rice at 1 dpi, some of them were later upregulated at 3 dpi (Fig. 6 and Table S6). These results suggest that XanA and Imp might implicate in various types of mechanisms that modulate rice lipid metabolism.

Metabolic pathways, such as secondary metabolism, S-assimilation, starch synthase, and amino acid synthesis and degradation, are not only necessary for normal growth and development but are also involved in the response to biotic and abiotic stresses in plants (Atkinson et al. 2013, Hu et al. 2018). In this study, most of the DEGs related to starch synthase, S-assimilation, tyrosine degradation (loc_os11g42510.1), cysteine synthesis and methionine synthesis were specifically downregulated in ∆*imp*-treated leaves, while DEGs related to tryptophan synthesis were specifically upregulated in ∆*xanA*-treated leaves both at 1 and 3 dpi (Fig. 6 and Table S6). Among secondary metabolites, phenylpropanoid, phenolics and terpenes are usually induced following pathogen infection and are believed to play important defensive roles in the plant kingdom (Taiz et al. 2014). Several genes related to phenylpropanoid pathways are also found to be highly expressed under biotic stress conditions, including *M. oryzae* and PXO99^A^ infection (Tariq et al. 2018, Tian et al. 2018). Here, our study showed that ∆*imp* infection suppressed the expression of most of the DEGs representing the “phenylpropanoids and phenolics” and “terpenes” at 1 dpi and 3 dpi compared with PXO99^A^ infection; however, the DEGs involved in phenolic metabolism pathways were downregulated at 1 d after ∆*xanA* infection and then upregulated at 3 d after ∆*xanA* infection (Fig. 6 and Table S6). These observations suggested that rice might utilize various defense strategies represented by differential expression of the genes related to the abovementioned metabolic pathways to cope with Imp- and XanA-mediated infection.

Plant hormones are signaling molecules that not only govern important plant physiological traits but are key players in plant-microbe interactions (Chanclud and Lacombe 2017). In the present study, the pathways of hormones such as auxin, brassinosteroid, ABA, ethylene, SA, and JA were significantly enriched and were differentially regulated in response to ∆*imp* and ∆*xanA* infection compared with PXO99A infection (Fig. 7 and Table S7). Tariq et al. found that a number of JA-, ethylene- and brassinosteroid-responsive genes in rice were downregulated after PXO99^A^ infection (Tariq et al. 2019). It was found in another study that several genes associated with ET hormones were induced after PXO99^A^ infection in the resistant rice CBB23 compared with those in the susceptible rice JG30 (Tariq et al. 2018). Interestingly, we found that ∆*imp* infection led to downregulated expression of most plant hormone-related genes, especially genes related to auxin, brassinosteroid, ethylene, and JA, while ∆*xanA* infection resulted in upregulated expression of most plant hormone-related genes, especially genes related to ethylene (Fig. 7 and Table S7). These results indicate that rice might employ different hormone signaling pathways to respond to ∆*imp* and ∆*xanA* infection. Pathogenesis-related proteins (PRs), which possess antimicrobial activities, are commonly used as signatures of SA-dependent systemic acquired resistance (Elsharkawy et al. 2013). Here, most of the genes encoding PRs and related proteins were found to be significantly upregulated in ∆*imp-*infected leaves compared with those in PXO99^A^-infected leaves, especially at 1 d after ∆*imp* infection. Additionally, two SA-related genes were found to be significantly upregulated at 1 d after ∆*imp* infection, whereas no DEGs associated with the SA pathway were detected in response to ∆*xanA* infection both at 1 and 3 dpi (Fig. 7 and Table S7), suggesting that the upregulation of SA-related genes (SA signaling pathway) might be the inducer of pathogenesis-related proteins in ∆*imp*-infected leaves.

Transcription factors (TFs) are important regulators of rice gene expression and play crucial roles in diverse physiological processes and initiation of stress and defense signaling (Samad et al. 2017). In this study, DEGs involved in different TF families, including AP2/EREBP, bZIP, WRKY, MYB and DOF, were detected. Among them, most AP2/EREBP genes were downregulated both at 1 and 3 d after ∆*imp* infection relative to those after PXO99^A^ infection and were only downregulated at 1 d after ∆*xanA* infection relative to those after PXO99^A^ infection (Fig. 7 and Table S7). It has been reported that AP2/EREBP TFs can act as positive regulators as well as negative regulators in resistance to pathogen infection, and the expression levels of several AP2/ERF TFs could be induced or repressed by various biotic stresses as well as abiotic stresses (Seo et al. 2015). MYB TFs have also been shown to have functions as both transcriptional activators and repressors in regulatory networks controlling phenylpropanoid metabolism and responses to biotic and abiotic stresses, e.g., AtMYB030, AtMYB060, AtMYB096 and HvMYB6 (Smita et al. 2015, Garner et al. 2016). Furthermore, several studies of the *Xoo*-rice interaction have observed that the expression of some rice MYB TFs, such as MYB51, R2R3-MYB and MYB4, were activated or upregulated in leaves at the early stage of PXO99^A^ infection (Tariq et al. 2019; Wang et al. 2019). In our study, all detected MYB genes were downregulated both at 1 and 3 dpi in ∆*xanA-*treated leaves relative to PXO99^A^-treated leaves, while the varied expression patterns of MYB-related genes were revealed at 1 and 3 dpi in ∆*imp-*treated rice leaves relative to those in PXO99^A^-treated leaves(Fig. 7 and Table S7), suggesting that XanA and Imp play crucial but distinct roles in modulation of MYB TF-mediated defense responses against PXO99^A^ infection. In plants, bZIP TFs, particularly those from the TGA family, have been demonstrated to have diverse roles in ABA and SA signaling and the response to abiotic/biotic stress (Alves et al. 2013), while WRKY TFs mainly act in SA/JA/ET-mediated signaling pathways as both positive and negative regulators of diverse biological processes, especially plant defense responses (Peng et al. 2016). Numerous studies have shown that most WRKY genes were transcriptionally induced by SA treatment or pathogen stresses, such as infection with *Xoo* and *M. oryzae*. Interestingly, our study found that most WRKY genes and bZIP genes were downregulated only in ∆*imp-*treated rice leaves relative to those in PXO99^A^-treated leaves at 1 dpi (Fig. 7 and Table S7), indicating the potential role of *Xoo* Imp in affecting WRKY- or bZIP-mediated defense responses at the early stage of infection.

A group of genes related to the cell wall, beta-glucanase, and proteolysis were also transcriptionally influenced in response to ∆*xanA* and ∆*imp* infection. Among them, most of the 1,3-beta-glucanase-related genes were specifically found to be downregulated in ∆*imp-*treated rice leaves both at 1 and 3 dpi (Fig. 7 and Table S7), indicating that Imp might enhance defense responses mediated by beta-glucanase in rice. 1,3-beta-glucanases are involved in diverse plant physiological processes, such as cell wall metabolism and plant defense, and various 1,3-beta-glucanase genes from plants, such as *Arabidopsis thaliana*, *Oryza sativa* and sugarcane, have been observed to be induced by pathogen attack in the early stage (Su et al. 2016). In contrast, Iglesias and Meins (Iglesias and Meins Jr. 2000) observed a reduction in systemic symptoms in a 1,3-beta-glucanase-deficient mutant of tobacco when infected by viruses (Iglesias and Meins Jr. 2000). The plant cell wall, consisting of cellulose microfibrils, hemicelluloses, pectic polysaccharides and proteins, not only provides a dynamic structure to support plant development but also acts as a battleground where plants directly encounter pathogens and subsequently activate defense signaling pathways (Houston et al. 2016). In this study, most DEGs associated with the cell wall were downregulated in ∆*imp-*treated rice leaves both at 1 and 3 dpi; however, in ∆*xanA-*treated rice leaves, most cell wall-related DEGs were downregulated at 1 dpi and then upregulated at 3 dpi (Fig. 7 and Table S7). To date, increasing evidence indicates that alterations in the the cell wall during biotic stress, especially cell wall damage or transcriptionally impairing cell wall-related genes, could trigger plant defense responses (Hamann 2012). For example, reduction of cellulose synthase (CESA3) or pectate lyase (PMR6) confers enhanced resistance to infection by certain pathogens (Vogel et al. 2002, Hamann 2012). Protease- and ubiquitin-dependent proteolysis have been shown to be widely involved in plant development and plant–pathogen interactions, and transcriptomic evidence has accumulated that proteolytic factors exhibit marked enrichment and increased expression in response to pathogen treatments (Pogany et al. 2015). Here, our work indicates that loss of *Xoo* Imp leads to downregulated expression of most proteolysis-related genes in infected leaves both at 1 and 3 dpi, while deficiency of *Xoo* XanA results in varied expression patterns of proteolysis-related genes and fewer DEGs at 1 and 3 dpi compared to those under ∆*imp* infection (Fig. 7 and Table S7).

Signaling networks/pathways including 14–3-3 protein-mediated signaling, G-protein signaling, MAP kinase signaling, calcium signaling and receptor kinase signaling are closely linked with plant functions in immunity and stress responses. In this study, one 14–3-3-like protein GF14 epsilon encoding gene (loc_os11g39540) and most genes involved in G-protein signaling and MAP kinase signaling (loc_os05g50560, loc_os04g35100 and loc_os05g49140) were found to be downregulated only in ∆*imp*-treated rice leaves at 1 dpi (Fig. 7 and Table S7), indicating their important roles at the initial infection stage. Among them, plant 14–3-3 proteins are recognized as mediators that interact with defense-related proteins or phosphorylated proteins, and there is evidence in rice that the GF14 genes were differentially induced during ETI elicited by *Xanthomonas oryzae* pv. *oryzae* (Manosalva et al. 2011). Plant MAPK cascades play fundamental roles in the transduction of extracellular stimuli and the establishment of resistance to pathogens (Wang et al. 2018). Surveys involving OsMAPK20 orthologs showed that the expression of LOC_Os05g49140 (OsMAPK20–5) from rice was downregulated in response to RBSDV infection (Ahmed et al. 2017), whereas GhMPK20 from cotton was significantly induced by *Fusarium oxysporum* and negatively regulated resistance through the MKK4-MPK20-WRKY40 cascade (Wang et al. 2018). Our data also showed that a variety of genes involved in calcium signaling and receptor kinase signaling were affected both by ∆*imp* infection and ∆*xanA* infection compared with the control (Fig. 7 and Table S7). Accumulated evidence has indicated that both calcium signaling and receptor kinase signaling could be modulated/activated upon pathogen attack and that the calcium signaling induced by MAMPs requires particular receptor-like kinases (Seybold et al. 2014). Notably, the vast majority of genes encoding receptor-like kinases were upregulated both at 1 and 3 dpi in ∆*imp-*treated rice leaves. In ∆*xanA-*treated rice leaves, two receptor kinase-related genes (loc_os03g17300 and loc_os12g34770) were downregulated at 1 dpi, and then three receptor kinase-related genes (loc_os05g44770, loc_os09g38830 and loc_os09g38834) were upregulated at 3 dpi. In addition, the majority of calcium signaling-related genes were downregulated both at 1 and 3 dpi in ∆*imp-*treated rice leaves, and only one calcium signaling-related gene (loc_os10g28240) was upregulated at 3 dpi (Fig. 7 and Table S7). These results suggest that XanA and Imp of *Xoo* have important but distinct roles in modulating signaling networks related to plant immunity.

## Conclusions

In this study, comparative proteomics analysis and pathogenicity tests revealed that 4 pathogenic-related proteins (XanA, Imp, RocF and MinD) of *Xoo* were continuously induced by host rice at 3, 6, and 12 h in an in vitro interaction system. Among them, two carbohydrate metabolism enzymes, XanA and Imp, were identified as novel virulence factors, and mutants of their encoding genes, Δ*xanA* and Δ*imp*, were almost avirulent on the susceptible rice IR24. Moreover, the RNA-seq analysis provided comprehensive information on a series of genes that were significantly differentially expressed in Δ*xanA*-treated and Δ*imp*-treated rice when compared to those in PXO99^A^-treated rice at 1 and 3 dpi. Through GO, KEGG pathway and MapMan analyses, the DEGs from the comparison of PXO99^A^ vs Δ*imp* were mainly identified to be involved in photosynthesis, signal transduction, transcription, oxidation-reduction, hydrogen peroxide catabolism, ion transport, phenylpropanoid biosynthesis and the metabolism of carbohydrates, lipids, amino acids, secondary metabolites, hormones, nucleotides and nitrogen, while the DEGs from the comparison of PXO99^A^ vs Δ*xanA* were predominantly associated with photosynthesis, signal transduction, oxidation-reduction, phenylpropanoid biosynthesis, cytochrome P450 and the metabolism of carbohydrates, lipids, amino acids, secondary metabolites and hormones. Although the DEGs and pathways affected by the Δ*imp* and Δ*xanA* treatments were not exactly the same, modulation of primary metabolism, secondary metabolism, photosynthesis and biotic stress pathways were common responses that were shared between Δ*imp-*treated rice and Δ*xanA-*treated rice. These results provide valuable insights into the molecular mechanism of pathogen infection strategies and plant immunity and reveal the potential functions of host-induced carbohydrate metabolism enzymes in *Xoo*-rice interactions.

## Methods

### Bacterial Strains, Plasmids, Primers and Culture Conditions

The details regarding the bacterial strains and plasmids used in this work are provided in Table S9. The primers used for mutant construction and qRT-PCR are listed in Table S10. The *Xoo* wild-type strain PXO99^A^ and its derivatives were cultured at 28 °C in liquid nutrient broth (NB) medium or NA (NB agar) plates (Qian et al. 2013a). *E. coli* DH5α used for plasmid construction was grown at 37 °C in Luria-Bertani (LB) medium or LB agar plates. When required, the corresponding medium was supplemented with antibiotics at the following concentrations for *E. coli* and *Xoo*: 100 μg/ml ampicillin (Amp), 50 μg/ml kanamycin (km), and 5 μg/ml gentamicin (Gm).

### Rice Leaf Extract Treatments, Induction Experiments and Preparation of Total Proteins from *Xoo* Cells

Rice leaf extract (RLX) for the in vitro assay system was prepared as described in a previously published method with minor modifications (Tahara et al. 2003; Kim et al. 2016). Briefly, 5- to 6-week-old leaves of the susceptible rice cultivar IR24 were harvested, washed several times with sterile water and ground into homogenate, which was used as rice leaf extract (RLX). Then, 5 g of RLX was macerated in 100 mL of NB medium, and the resulting +RLX (NB plus RLX) medium was centrifuged and filtered using 0.22 μm membranes (Millipore, Bedford, MA, USA). The experiments to induce differential proteins were carried out by growing *X. oryzae* pv. *oryzae* cells in the -RLX (NB medium) and + RLX induction medium. The *Xoo* cells (OD_600_ ≈ 0.3) in 180 mL of NB medium were pelleted and resuspended in 1.2 mL of the same medium, and aliquots of 200 μL were used to inoculate 30 mL of NB and induction medium and then cultured for 3, 6 and 12 h before sampling.

Total proteins from *Xoo* cells were prepared as described previously with minor modifications (Zhao et al. 2011). Briefly, sample cells were harvested by centrifugation, pelleted and resuspended in 20 mL washing buffer (50 mmol/L Tris-HCI, pH 7.2), and repeatedly centrifuged at 3000 rpm for 10 min at 4 °C twice. Subsequently, the pellet was resuspended in 10 mL Alklysis buffer containing protease inhibitor cocktail (St. Louis, MO, USA) and 1 mM PMSF and fragmented by ultrasonication. The lysate was centrifuged at 12,000 rpm for 30 min at 4 °C. The supernatant was collected, and the total protein of each sample was purified using a protein clean-up kit (GEHealthcare Life Sciences, USA). Isoelectric focusing (IEF) buffer containing 7 M urea, 2 M thiourea, 4% CHAPS, 40 mM DTT and 2% (vol/vol) immobilized pH gradient (IPG) buffer, at a pH of 4 to 7, was used to dissolve the protein sample pellets. The concentration of each protein was determined using the QuickStart Bradford Protein Assay Kit (Bio-Rad Laboratories, Hercules, CA, USA). Protein samples from three biological replicate experiments were then stored at − 80 °C for later use in 2-DE (two-dimensional gel electrophoresis).

### 2-DE and MALDI-TOF MS Analysis

The methods used for 2-DE and MALDI-TOF MS analysis were as described in our earlier works (Zhao et al. 2011; Qian et al. 2013a). The protein samples were adjusted to the appropriate loading amount (200 μg) and separated by 2-DE. Silver staining was performed according to a published procedure (Zhao et al. 2011). The comparative analysis of the resulting images was performed using PDQuest v7.2 software (Bio-Rad Laboratories, Hercules, CA, USA). The spots of differentially expressed proteins were excised manually from the 2-D gels. Destaining of silver-stained gels and in-gel trypsin digestion were performed as previously described (Calhoun et al. 2010). The masses of tryptic-digested peptide were determined using a MALDI-TOF-TOF 4700 mass spectrometer (Applied Biosystems, Foster City, CA, USA). The resulting data were analyzed using the Data Explorer software package (Applied Biosystems), and the identification was performed with the MASCOT program (Matrix Science, London, UK) using the probability-based Mowse score and a threshold of *P* < 0.05. Similarity searches were performed using BLAST and the genome database of strain PXO99^A^ (Salzberg et al. 2008).

### Generation of Gene Deletion Mutants and Complemented Strains in PXO99^A^

To generate an in-frame deletion mutant of the differentially expressed protein-coding genes in PXO99^A^, allelic homologous recombination was applied using the suicide vector pK18mobsacB as described previously (Qian et al. 2013b). Briefly, two flanking regions of the target gene were amplified with primers **(**Table S10**)** and ligated into pK18mobsacB. The resulting recombinant vectors for each target gene were validated by sequencing and introduced into PXO99^A^ cells via electroporation using a Bio-Rad Micropulser. Transformants were selected on NANS (NA without sucrose) plates containing 50 μg/ml Km for the first crossover event. Positive colonies were then plated on NA plates containing 10% (w/v) sucrose to screen for a second crossover event. After two rounds of screening, the resulting mutants were confirmed by PCR analysis. For complementation, each target gene with its predicted promoter region was amplified by PCR with specific primer sets (Table S10) and cloned into the broad-host-range vector pUFR047 (Andrade et al. 2014). The resulting plasmid was then transferred into the corresponding mutant via electroporation to generate the complemented strains.

### Plant Materials and Pathogenicity Assays

The rice plants were grown at 22–30 °C in a greenhouse of Jiangsu Academy of Agricultural Science, Nanjing, China. Pathogenicity assays were performed on the *Xoo*-susceptible rice cultivar IR24 using the standard leaf-clipping method as previously described (Yang and Bogdanove 2013). Briefly, inoculations were performed by immersing scissors in freshly prepared suspensions of *Xoo* strains in sterile water at a concentration of OD_600_ = 0.5 and clipping approximately 2 cm from the tips of the uppermost leaves of 5- to 6-week-old rice plants. The lesion lengths were measured 16 dpi, and representative images of infected rice leaves were photographed. At least 9 leaves were inoculated with each tested strain in each replicate. The biological experiments were performed three times.

### Growth Assays

*X*. *oryzae* pv. *oryzae* strains were grown overnight in NB medium at 28 °C with shaking at 200 rpm. The optical densities of the cultures were adjusted to OD_600_ = 1.0 and diluted 1: 100 in 30 ml of fresh NB medium. Growth curves were monitored by measuring the OD_600_ every 6 h after inoculation, and all inoculated samples were grown at 28 °C until the stationary phase was achieved. Three biological experiments were performed.

### Preparation of *Xoo*-Infected Rice Samples for RNA Sequencing

The *Xoo*-infected rice samples were prepared for RNA sequencing by previously described methods (Yu et al. 2014; Zhang et al. 2015). The overnight cultured cells of *Xoo* strains were collected by centrifugation and resuspended in sterile water at a concentration of OD_600_ = 0.5. Four-centimeter-long leaf tips from IR24 rice inoculated with PXO99^A^ and the mutants of host-induced virulence genes were dissected at 1 and 3 dpi. Three biological replicates of the leaf samples were collected for each treatment at each time point. Samples inoculated with PXO99^A^, Δ*xanA* and Δ*imp* at 1 dpi were collected and named PXO99^A^_1d, *xanA*_1d and *imp*_1d, while samples collected at 3 dpi were named PXO99^A^_3d, *xanA*_3d and *imp*_3d. All samples were immediately frozen in liquid nitrogen after collection and stored at − 80 °C.

### RNA Extraction, Illumina Sequencing, and Transcriptome Data Analysis

Total RNA was extracted from each rice sample using TRIzol Reagent (TaKaRa, Dalian, China) and treated with RNase-free DNase according to the manufacturer’s instructions. The quality of extracted RNA samples was assessed using an Agilent 2100 Bioanalyzer (Agilent Technologies). The mRNA enrichment and cDNA libraries were constructed according to Yu et al. (Yu et al. 2014). Paired-end sequencing (2 × 150 bp) was performed using the Illumina HiSeq2000 platform in accordance with the manufacturer’s protocol (Illumina, San Diego CA, USA). The raw paired-end reads containing the adapter or poly-N or low-quality reads were cleaned for quality control using SeqPrep (https://github.com/jstjohn/SeqPrep) and Sickle (https://github.com/najoshi/sickle). The high-quality clean reads were mapped to the rice genome of MSU RGAP (http://rice.plantbiology.msu.edu) using TopHat2 (http://ccb.jhu.edu/software/tophat/index.shtml) and assembled with Cufflinks (http://cole-trapnell-lab.github.io/cufflinks/). The gene expression in each library was calculated and compared based on their fragments per kilobase of exon per million fragments mapped (FPKM) values. DESeq2 (Love et al. 2014) was applied to identify DEGs between two samples, and genes with a BH (Benjamini/Hochberg) corrected *p*-value < 0.05 and |log2 (fold change)| ≥ 1 were considered significantly differentially expressed.

### Bioinformatic Analyses and Functional Annotation of DEGs

The bioinformatic analyses and functional annotation of DEGs were conducted using MapMan (Thimm et al. 2004) and the i-Sanger platform (http://www.i-sanger.com/) provided by Shanghai Majorbio Biopharm Technology Co., Ltd. Briefly, for MapMan-based analysis, the DEGs from each comparison were uploaded to the MapMan tool, and the corresponding graphical representations were generated to visualize the expression changes of individual genes involved in biotic stress response and metabolic pathways. Based on the GO (Gene Ontology) (Ashburner et al. 2000), EggNOG (Evolutionary genealogy of genes: Nonsupervised Orthologous Groups) (Huerta-Cepas et al. 2016), and KEGG (Kyoto Encyclopedia of Genes and Genomes, http://www.genome.jp/kegg/) databases, the biological processes, functional categorizations and pathway annotations of all DEGs were characterized, and terms or pathways with corrected *P* values less than 0.05 were considered significantly enriched.

### Quantitative Real-Time PCR (qRT-PCR) Assay

The expression patterns of target genes were detected or verified by qRT-PCR as described previously (Zhang et al. 2015; Tariq et al. 2019). Briefly, independent RNA samples of IR24 rice were prepared following the same protocols as described for the RNA-Seq and RLX treatment assays. Gene-specific primers were designed to amplify sequences 80–150 bp in length from the rice genome (MSU RGAP, http://rice.plantbiology.msu.edu/) using Primer Express 3.0 (Applied Biosystems, Life Technologies). Total RNA was isolated using TRIzol Reagent (TaKaRa, Dalian, China), and cDNA was then synthesized from each RNA sample using the TransScript All-in-One First-Strand cDNA Synthesis SuperMix Kit (TransGen Biotech, Beijing, China) according to the manufacturer’s instructions. All qRT-PCR analyses were carried out on a QuantStudio™ 6 Flex Real-Time PCR System (Applied Biosystems, Foster City, CA, USA) using TransStart Top Green qPCR SuperMix (TransGen Biotech, Beijing, China). The relative expression levels of the selected genes were calculated with the 2^–ΔΔCT^ method using actin and eEF1a as the endogenous controls when analyses were required. The experiments were performed three times, and each experiment involved three replicates.

### DAB Staining and H_2_O_2_ Accumulation Assays

For observation of the water-soaked/hypersensitive reaction, leaves of 5- to 6-week-old IR24 rice were infiltrated with different *Xoo* suspensions with an optical density at 600 nm (OD_600_) of 0.5 using a needleless syringe, as previously described (Streubel et al. 2013, Yang and Bogdanove 2013). The water-soaked symptoms were scored and photographed 3 dpi. For H_2_O_2_ accumulation assays, rice leaves infiltrated with different *Xoo* suspensions at the indicated time points (1 and 3 dpi) were examined by 3,3′-diaminobenzidene (DAB) staining following the methods described previously (Girija et al. 2017, Sathe et al. 2019). Then, H_2_O_2_ accumulation was visualized by visible light microscopy using a 10× objective. To measure H_2_O_2_ levels in rice leaves, a Hydrogen Peroxide Assay Kit (Solarbio, Beijing, China) was used according to the instruction manual. All the rice inoculations with *Xoo* were biologically repeated at least three times, and each involved three replicates.

### Statistical Analysis

All data were analyzed by using SPSS v.19.0 (SPSS Inc., Chicago, IL, USA). Significant differences in lesion lengths, bacterial phenotypes and gene expressions among different strains were determined via the hypothesis test of percentages (t-test) (*P* < 0.05).

## Supplementary Informations

**Additional file 1: Table S1.** Detailed information of DEGs from four pairwise comparisons.

**Additional file 2: Table S2.** Gene ontology (GO) annotations of DEGs from four pairwise comparisons.

**Additional file 3: Table S3.** COG annotations of DEGs from four pairwise comparisons.

**Additional file 4: Table S4.** KEGG pathway enrichment analysis of DEGs from four pairwise comparisons.

**Additional file 5: Table S5.** GO enrichment analysis of DEGs from four pairwise comparisons.

**Additional file 6: Table S6.** MapMan analysis of DEGs involved in different metabolic pathways.

**Additional file 7: Table S7.** MapMan analysis of DEGs involved in biotic stress pathway.

**Additional file 8: Table S8.** DEGs from four pairwise comparisons used for venn diagram analysis.

**Additional file 9: Table S9.** Bacterial strains and plasmids used in this study.

**Additional file 10: Table S10.** Primers used in this study.

**Additional file 11: Figure S1.** Growth curves of wild type strain PXO99^A^, the mutant strains Δ*xanA*, Δ*imp*, Δ*rocF*, Δ*minD*, Δ*bfr* and their complemented strains in NB medium. **A**-**E**. All tested strains were cultivated at 28 °C with shaking at 220 rpm. Bacterial growth was determined by measuring the OD_600_ against the medium blank every 6 h after inoculation. Values are the means ± SD from three independent experiments. Δ*xanA*, the *xanA* deletion mutant; Δ*xanA*(*xanA*), the complemented strain of Δ*xanA*; Δ*imp*, the *imp* deletion mutant; Δ*imp*(*imp*), the complemented strain of Δ*imp*; Δ*rocF*, the *rocF* deletion mutant; Δ*rocF*(*rocF*), the complemented strain of Δ*rocF*; Δ*minD*, the *minD* deletion mutant; Δ*minD*(*minD*), the complemented strain of Δ*minD*; Δ*bfr*, the *bfr* deletion mutant; Δ*bfr*(*bfr*), the complemented strain of Δ*bfr*.

**Additional file 12: Figure S2.** GO enrichment analysis of DEGs from comparison groups PXO99^A^ vs Δ*xanA* (1d), PXO99^A^ vs Δ*xanA* (3d), PXO99^A^ vs Δ*imp* (1d) and PXO99^A^ vs Δ*imp* (3d). Histogram of the top 41 significantly enriched GO subcategories with the highest representation of the DEGs. These subcategories were further summarized into three main GO categories: biological process, molecular function, and cellular component. The names of the GO subcategories are listed along the x-axis. The y-axis indicates the number of enriched genes in different comparison groups. The degree of GO enrichment is represented by the FDR value. Asterisks indicate significant enrichment (*FDR < 0.05). The detailed information is shown in Table S5.

**Additional file 13: Figure S3.** Venn diagram showing the number of unique or overlapped rice DEGs between different pairwise comparisons. A. Distribution of unique or overlapped rice DEGs from different comparison groups at the same inoculation time points. B. Distribution of unique or overlapped rice DEGs from the same comparison groups at different inoculation time points. Detailed information is presented in Table S8.

**Additional file 14: Figure S4.** MapMan visualization of the DEGs involved in photosynthesis pathway at different time points. In each comparison group, DEGs with |log2 (fold change)| ≥ 1 were imported into MapMan software. The gray circles indicates no differentially expressed genes matched in this process. The red and blue squares attached in each photosynthesis pathway represent up- and down-regulated genes, respectively. The color intensity represents gene expression level (log2 ratio mutant/PXO99^A^), as indicated by the color scale. The detailed information is shown in Table S6.

## Data Availability

All relevant data are presented in the additional files. The RNA-Seq raw data obtained in this article have been deposited in NCBI’s Sequence Read Archive (SRA) and are accessible through SRA Series accession number PRJNA661715.
