# Supplementary material for: Proteomic and Transcriptomic Analyses Provide Novel Insights into the Crucial Roles of Host-Induced Carbohydrate Metabolism Enzymes in Xanthomonas oryzae pv. oryzae Virulence and Rice-Xoo Interaction
Source: Rice (N Y). 2021 Jun 26;14:57. doi: 10.1186/s12284-021-00503-x (PMC8236019; doi:10.1186/s12284-021-00503-x)
Supplement: Supplementary file 9 — Additional file 9: Table S9. Bacterial strains and plasmids used in this study. [file 12284_2021_503_MOESM9_ESM.docx]

**SUPPLEMENTARY Table S9. Bacterial strains and plasmids used in this study.**

| **Strains or plasmids** | **Characteristics^a^** | **Source** |
| --- | --- | --- |
| **Strains** |  |  |
| ***Escherichia coli*** |  |  |
| DH5a | *F-, φ80dlacZ∆M15, ∆(lacZYA-argF)U169, deoR, recA1, endA1, hsdR17(rk-,mk+), phoA, supE44, λ-, thi-1, gyrA96* | TransGen Biotech |
| ***Xanthomonas oryzae* pv*. oryzae*** |  |  |
| PXO99^A^ | Philippine race 6; Wild-type strain (WT) |  |
| Δ*xanA* | *xanA* in-frame deletion mutant of strain PXO99^A^ | This study |
| Δi*mp* | *imp* in-frame deletion mutant of strain PXO99^A^ | This study |
| Δ*rocF* | *rocF* in-frame deletion mutant of strain PXO99^A^ | This study |
| Δ*minD* | *minD* in-frame deletion mutant of strain PXO99^A^ | This study |
| Δ*bfr* | *bfr* in-frame deletion mutant of strain PXO99^A^ | This study |
| Δ*xanA*(*xanA*) | ∆*xanA* harboring plasmid pUFR047-*xanA* (Genetic complementary strain); Gm^R^, Amp^R^ | This study |
| Δ*imp*(*imp*) | ∆*imp* harboring plasmid pUFR047-*imp* (Genetic complementary strain); Gm^R^, Amp^R^ | This study |
| Δ*rocF*(*rocF*) | ∆*rocF* harboring plasmid pUFR047-*rocF* (Genetic complementary strain); Gm^R^, Amp^R^ | This study |
| Δ*minD*(*minD*) | ∆*minD* harboring plasmid pUFR047-*minD* (Genetic complementary strain); Gm^R^, Amp^R^ | This study |
| Δ*bfr*(*bfr*) | ∆*bfr* harboring plasmid pUFR047-*bfr* (Genetic complementary strain); Gm^R^, Amp^R^ | This study |
| **Plasmids** |  |  |
| pK18mob*sacB* | Km^R^, oriT(RP4), *sacB*, *lacZ*alpha, Plac, Pmbi, Mobilization and counter selection | Qian, Liu et al. 2013 |
| pK18-*xanA* | Km ^R^; pK18mob*sacB* with two *xanA* flanking fragments | This study |
| pK18-*imp* | Km^R^; pK18mob*sacB* with two *imp* flanking fragments | This study |
| pK18-*rocF* | Km^R^; pK18mob*sacB* with two *rocF* flanking fragments | This study |
| pK18-*minD* | Km^R^; pK18mob*sacB* with two *minD* flanking fragments | This study |
| pK18-*bfr* | Km^R^; pK18mob*sacB* with two *bfr* flanking fragments | This study |
| pUFR047 | Broad-host-range expression vector; IncW, Gm^R^, Amp^R^, Mob^+^, Mob(p), lacZα^+^, Par^+^ | Andrade, Farah et al. 2014 |
| pUFR047-*xanA* | pUFR047 carrying *xanA* gene with its native promoter region; Gm^R^, Amp^R^ | This study |
| pUFR047-*imp* | pUFR047 carrying *imp* gene with its native promoter region; Gm^R^, Amp^R^ | This study |
| pUFR047-*rocF* | pUFR047 carrying *rocF* gene with its native promoter region; Gm^R^, Amp^R^ | This study |
| pUFR047-*minD* | pUFR047 carrying *minD* gene with its native promoter region; Gm^R^, Amp^R^ | This study |
| pUFR047-*bfr* | pUFR047 carrying *bfr* gene with its native promoter region; Gm^R^, Amp^R^ | This study |

^a^ Km^R^, Gm^R^ Amp^R^, Tet^R^, Chlo^R^ = kanamycin, gentamicin, ampicillin, tetracycline, chloramphenicol respectively.
