# Supplementary material for: Proteomic and Transcriptomic Analyses Provide Novel Insights into the Crucial Roles of Host-Induced Carbohydrate Metabolism Enzymes in Xanthomonas oryzae pv. oryzae Virulence and Rice-Xoo Interaction
Source: Rice (N Y). 2021 Jun 26;14:57. doi: 10.1186/s12284-021-00503-x (PMC8236019; doi:10.1186/s12284-021-00503-x)
Supplement: Supplementary file 10 — Additional file 10: Table S10. Primers used in this study. [file 12284_2021_503_MOESM10_ESM.docx]

**SUPPLEMENTARY Table S10. Primers used in this study.**

| Primer | Sequence (5’ to 3’)^a^ | Purpose/Description | Source |
| --- | --- | --- | --- |
| **For** **mutant construction** | | | |
| xanA-1F | CGGGATCCTCTTGCGGATCTGTCGG | Amplify a 452 bp upstream homologue arm of xanA | This study |
| xanA-1R | CCCAAGCTTGGCCAAGTCCTCGTTGAGTTC |  | This study |
| xanA-2F | CCCAAGCTTCGGCGAGATCAACTTCAAGGT | Amplify a 557 bp downstream homologue arm of xanA | This study |
| xanA-2R | GCTCTAGAGGTGTTGCGGCCTTTGG |  | This study |
| imp-1F | CGGGATCCAGTCAGTGCGGTAACGCAAGA | Amplify a 329 bp upstream homologue arm of imp | This study |
| imp-1R | CCCAAGCTTCCGGCAACATCAAGATCAGC |  | This study |
| imp-2F | CCCAAGCTTGCGCTTGAGTTCCTTGATGAT | Amplify a 276 bp downstream homologue arm of imp | This study |
| imp-2R | GCTCTAGAGCTGTTTGGCCGTAGATGGT |  | This study |
| rocF-1F | CGGGATCCAAACGATTGATCACGGCACT | Amplify a 725 bp upstream homologue arm of rocF | This study |
| rocF-1R | CCCAAGCTTACATCGTCGAGCTCAACCC |  | This study |
| rocF-2F | CCCAAGCTTAATCAGGGAAACCGGCACGTA | Amplify a 663 bp downstream homologue arm of rocF | This study |
| rocF-2R | GCTCTAGACGCTTCGCATGAAAGACGTT |  | This study |
| minD-1F | CGGGATCCGCTTACTTGTCGCCGTCTTCC | Amplify a 323 bp upstream homologue arm of minD | This study |
| minD-1R | CCCAAGCTTCCCGATGCGATTCACATCCG |  | This study |
| minD-2F | CCCAAGCTTACTTTCTTGCCGCGCTTT | Amplify a 411 bp downstream homologue arm of minD | This study |
| minD-2R | GCTCTAGAAGAACTGCGACCTGACCGTA |  | This study |
| bfr-1F | CGGGATCCGCGTTTCAGGAAATAAAACTGCT | Amplify a 365 bp upstream homologue arm of bfr | This study |
| bfr-1R | CCCAAGCTTGCTGTACCAGACCTCGAAG |  | This study |
| bfr-2F | CCCAAGCTTCCTTTCATGGTGTGCTCCAACGA | Amplify a 475 bp downstream homologue arm of bfr | This study |
| bfr-2R | GCTCTAGACTTGTCCAGGTCTACGGCCTT |  | This study |
| **For construction of genetic complementary strain** | | | |
| CΔ*xanA*-F | CCCAAGCTTGAAATTGCGTGCCGTCTT | Amplify a 2032 bp fragment containing *xanA* gene and its predicted promoter | This study |
| CΔ*xanA*-R | GGGGTACCTCAGCCGCGCAGCAGGTTGGA |  | This study |
| CΔ*imp*-F | CGGAATTCTCAGAACGTGGCGTCGAACTCGC | Amplify a 1065 bp fragment containing *imp* gene and its predicted promoter | This study |
| CΔ*imp*-R | CCCAAGCTTACCAGCACCAGGCGCGAGAC |  | This study |
| CΔ*rocF*-F | CGGAATTCTCAATCCCGCATCAGCGTCGATT | Amplify a 1237 bp fragment containing *rocF* gene and its predicted promoter | This study |
| CΔ*rocF*-R | CGGGGTACCCCTGTTGCTTGATGGCCTGA |  | This study |
| CΔ*minD*-F | CGGAATTCTTAGCCTCCGAACAGCTTGCTGA | Amplify a 954 bp fragment containing *minD* gene and its predicted promoter | This study |
| CΔ*minD*-R | CGGGGTACCCATCGCTGGCAACTACAAGG |  | This study |
| CΔ*bfr*-F | CGGAATTCTCAACCGGCAGCGTGGCCCTTGT | Amplify a 833 bp fragment containing *bfr* gene and its predicted promoter | This study |
| CΔ*bfr*-R | CGGGGTACCCGCATCGAGCTGATTTGTG |  | This study |
| **For RNA-Seq data validation** | | | |
| LOC_Os01g61880.1F | GAGAGCTTCGGCAAGTGCAT | Respiratory burst oxidase | This study |
| LOC_Os01g61880.1R | CCTTGAGCTGCTCCTTGGTG |  | This study |
| LOC_Os04g49350.1F | GGACCGGTGCATGGATTTGT | Pentatricopeptide repeat domain containing protein | This study |
| LOC_Os04g49350.1R | TACAGCCTCCTGTCCAGTCG |  | This study |
| LOC_Os03g06630.1F | TCCCACGCTTCTTCAAGCAC | Heat stress transcription factor | This study |
| LOC_Os03g06630.1R | AGATGCCTCTGGCCTCTCAG |  | This study |
| LOC_Os09g28390.1F | CCAAGCTCGAGATGCTCGTC | Abscisic acid 8'-hydroxylase 3 | This study |
| LOC_Os09g28390.1R | ATGCTTGGGCACAGGGAATG |  | This study |
| LOC_Os08g33820.1F | CAGGCGATAACGGGTTCGAC | Chlorophyll A-B binding protein | This study |
| LOC_Os08g33820.1R | CCGATCTTCGTCAGCACCTC |  | This study |
| LOC_Os04g41960.1F | CACTACGCCAGGTACAGGGA | NADP-dependent oxidoreductase | This study |
| LOC_Os04g41960.1R | TCCTCCACGTACGCGATCTT |  | This study |
| LOC_Os02g41510.1F | CCTCGGAGGAGGAGTTCCAG | R2R3-type MYB transcription factor | This study |
| LOC_Os02g41510.1R | TCATCTCCATCCCGGAGTCG |  | This study |
| LOC_Os03g09220.1F | TCTGAGGTCGGTGTCGAGTC | BTH-induced protein phosphatase 2C 1 | This study |
| LOC_Os03g09220.1R | GTTTCCATCCCGTCCACACC |  | This study |
| LOC_Os09g11480.1F | CCACAAGGCAAGGACCAACT | Ethylene-responsive transcription factor | This study |
| LOC_Os09g11480.1R | CGTCGAGCAGGAAGCAGAAC |  | This study |
| eEF1a-F | TTTCACTCTTGGTGTGAAGCAGAT | Elongation factor Tu | This study |
| eEF1a-R | GACTTCCTTCACGATTTCATCGTAA |  | This study |
| actin-F | TCTTACGGAGGCTCCACTTAAC | Actin | This study |
| actin-R | TCCACTAGCATAGAGGGAAAGC |  | This study |

References cited in Supplementary materials

Andrade, M. O., C. S. Farah and N. Wang (2014). "The post-transcriptional regulator rsmA/csrA activates T3SS by stabilizing the 5' UTR of hrpG, the master regulator of hrp/hrc genes, in Xanthomonas." PLoS Pathog 10(2): e1003945.

Qian, G. L., C. H. Liu, G. C. Wu, F. Q. Yin, Y. C. Zhao, Y. J. Zhou, Y. B. Zhang, Z. W. Song, J. Q. Fan, B. S. Hu and F. Q. Liu (2013). "AsnB, regulated by diffusible signal factor and global regulator Clp, is involved in aspartate metabolism, resistance to oxidative stress and virulence in Xanthomonas oryzae pv. oryzicola." Molecular Plant Pathology **14**(2): 145-157.
