# Supplementary figures and images for: Proteomic and Transcriptomic Analyses Provide Novel Insights into the Crucial Roles of Host-Induced Carbohydrate Metabolism Enzymes in Xanthomonas oryzae pv. oryzae Virulence and Rice-Xoo Interaction
Source: Rice (N Y). 2021 Jun 26;14:57. doi: 10.1186/s12284-021-00503-x (PMC8236019; doi:10.1186/s12284-021-00503-x)

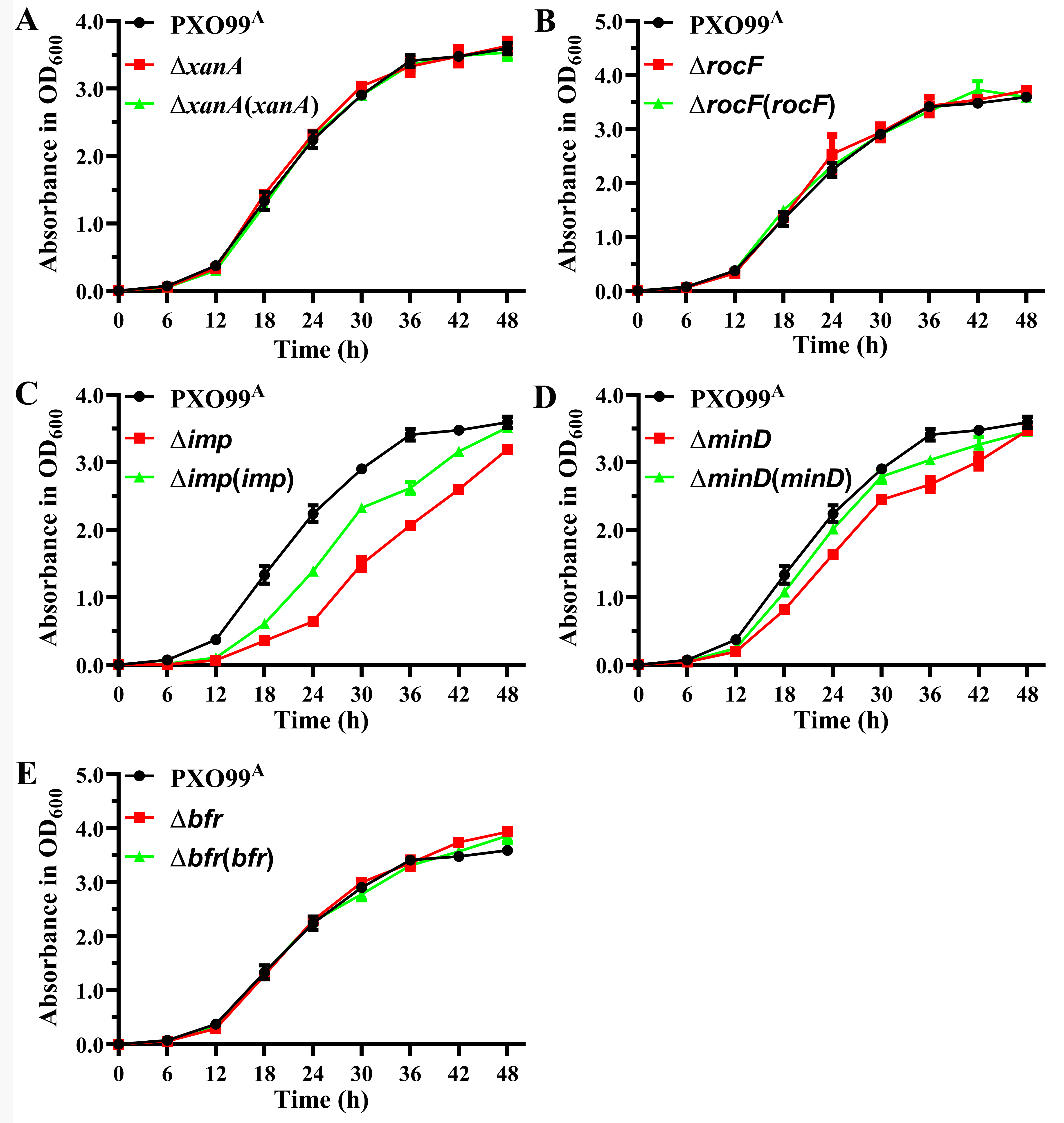

Supplement: Supplementary file 11 — Additional file 11: Figure S1. Growth curves of wild type strain PXO99A, the mutant strains ΔxanA, Δimp, ΔrocF, ΔminD, Δbfr and their complemented strains in NB medium. A-E. All tested strains were cultivated at 28 °C with shaking at 220 rpm. Bacterial growth was determined by measuring the OD600 against the medium blank every 6 h after inoculation. Values are the means ± SD from three independent experiments. ΔxanA, the xanA deletion mutant; ΔxanA(xanA), the complemented strain of ΔxanA; Δimp, the imp deletion mutant; Δimp(imp), the complemented strain of Δimp; ΔrocF, the rocF deletion mutant; ΔrocF(rocF), the complemented strain of ΔrocF; ΔminD, the minD deletion mutant; ΔminD(minD), the complemented strain of ΔminD; Δbfr, the bfr deletion mutant; Δbfr(bfr), the complemented strain of Δbfr. [file 12284_2021_503_MOESM11_ESM.tif]

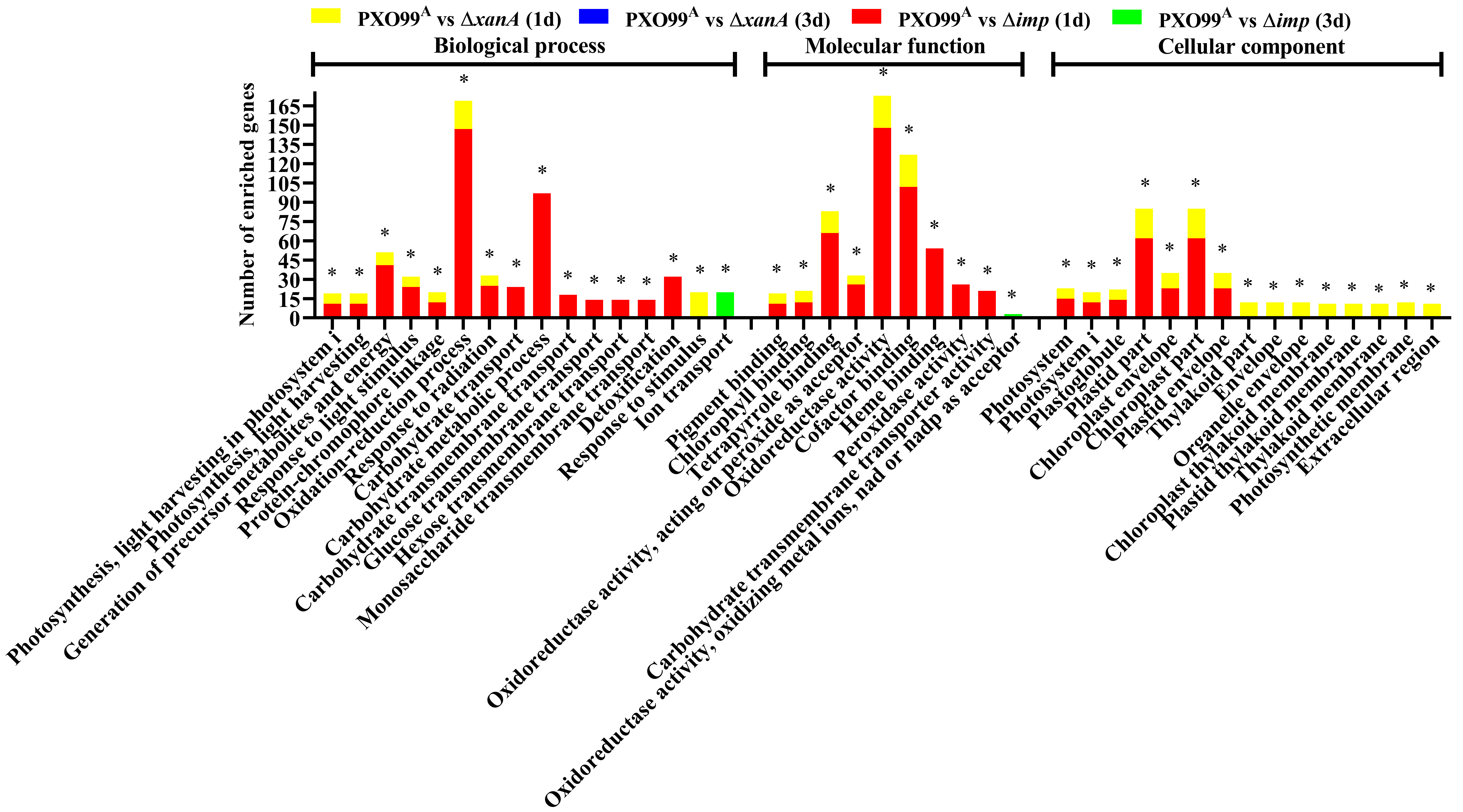

Supplement: Supplementary file 12 — Additional file 12: Figure S2. GO enrichment analysis of DEGs from comparison groups PXO99A vs ΔxanA (1d), PXO99A vs ΔxanA (3d), PXO99A vs Δimp (1d) and PXO99A vs Δimp (3d). Histogram of the top 41 significantly enriched GO subcategories with the highest representation of the DEGs. These subcategories were further summarized into three main GO categories: biological process, molecular function, and cellular component. The names of the GO subcategories are listed along the x-axis. The y-axis indicates the number of enriched genes in different comparison groups. The degree of GO enrichment is represented by the FDR value. Asterisks indicate significant enrichment (*FDR < 0.05). The detailed information is shown in Table S5. [file 12284_2021_503_MOESM12_ESM.tif]

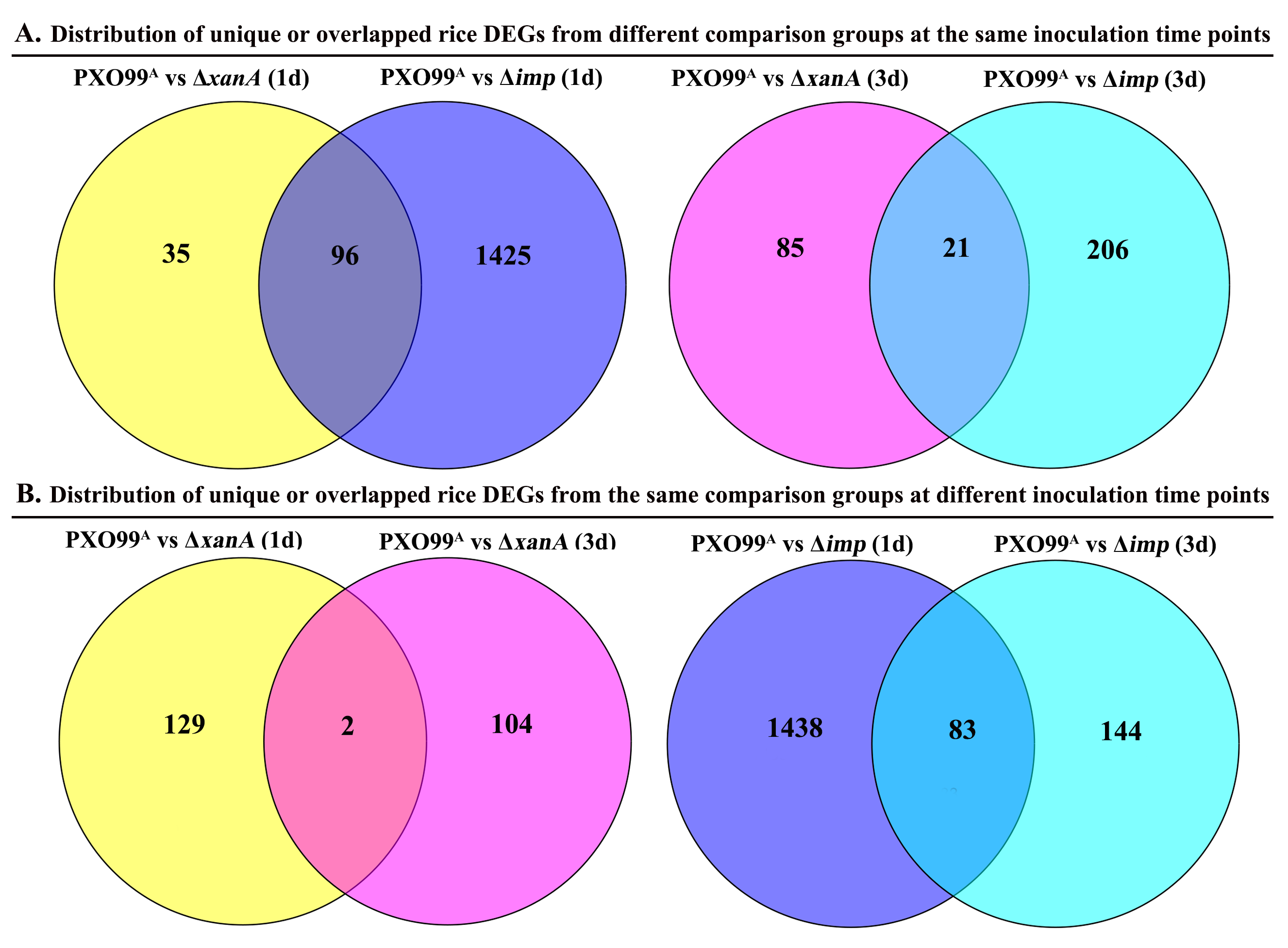

Supplement: Supplementary file 13 — Additional file 13: Figure S3. Venn diagram showing the number of unique or overlapped rice DEGs between different pairwise comparisons. A. Distribution of unique or overlapped rice DEGs from different comparison groups at the same inoculation time points. B. Distribution of unique or overlapped rice DEGs from the same comparison groups at different inoculation time points. Detailed information is presented in Table S8. [file 12284_2021_503_MOESM13_ESM.tif]

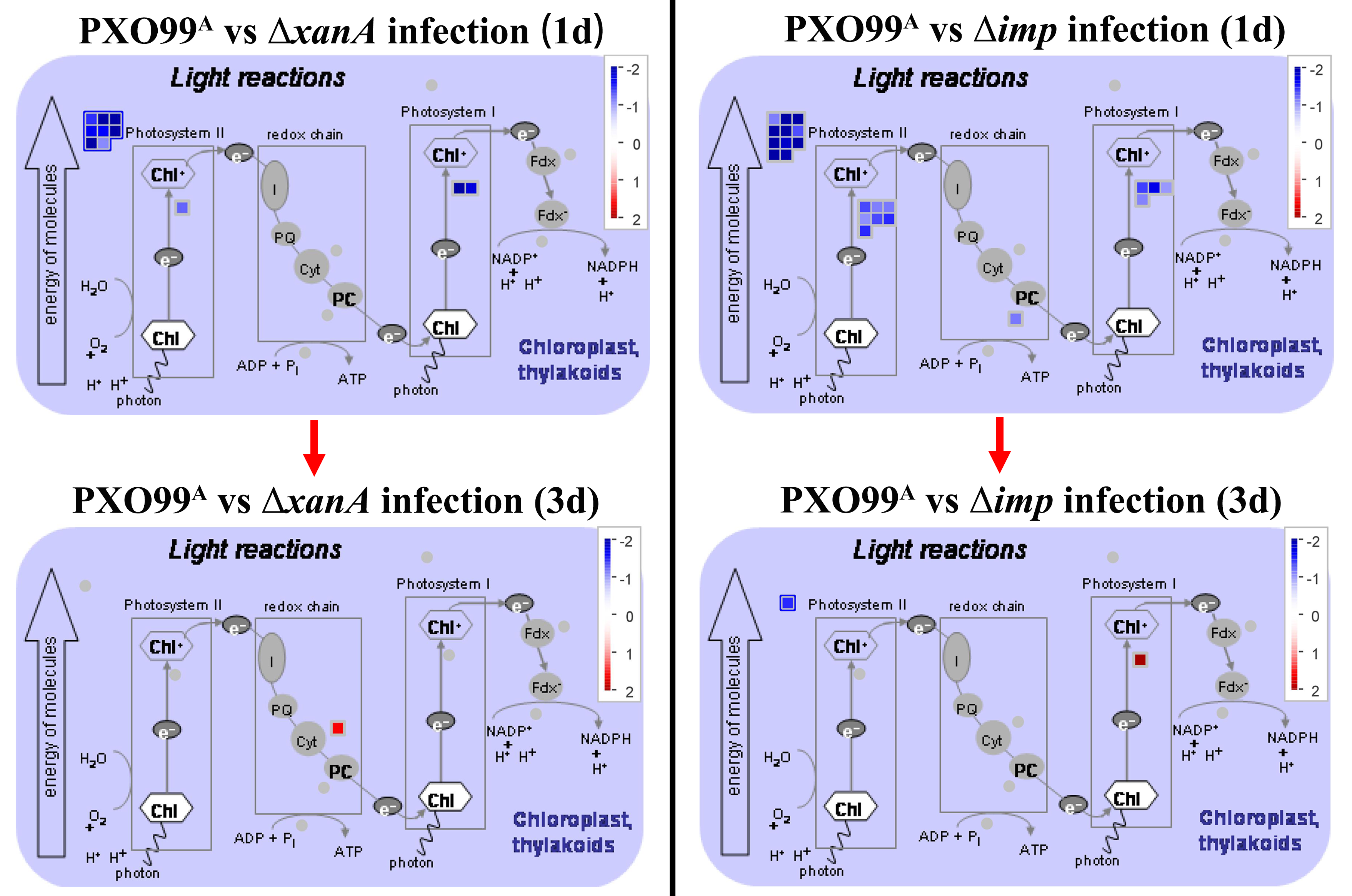

Supplement: Supplementary file 14 — Additional file 14: Figure S4. MapMan visualization of the DEGs involved in photosynthesis pathway at different time points. In each comparison group, DEGs with |log2 (fold change)| ≥ 1 were imported into MapMan software. The gray circles indicates no differentially expressed genes matched in this process. The red and blue squares attached in each photosynthesis pathway represent up- and down-regulated genes, respectively. The color intensity represents gene expression level (log2 ratio mutant/PXO99A), as indicated by the color scale. The detailed information is shown in Table S6. [file 12284_2021_503_MOESM14_ESM.tif]
